# EAACI/ESCD Skin Allergy Meeting 2017 (SAM 2017)

**DOI:** 10.1186/s13601-017-0184-5

**Published:** 2017-12-15

## Thursday, 27 April 2017

### O01 Methylisothiazolinone contact allergy: a real outbreak

#### Luis Amaral^1^, Emidio Silva^2^, Marcio Oliveira^3^, Ana Paula Cunha^4^

##### ^1^Serviço de Imunoalergologia, Centro Hospitalar de São João E.P.E., Porto, Portugal; ^2^Serviço de Medicina do Trabalho e Saúde Ocupacional, Centro Hospitalar do Baixo Vouga E.P.E., Aveiro, Portugal; ^3^Serviço de Saúde Ocupacional, Centro Hospitalar de São João E.P.E., Porto, Portugal; ^4^Serviço de Dermatologia, Centro Hospitalar de São João E.P.E., Porto, Portugal

###### **Correspondence:** Luis Amaral - luis.m.amaral@gmail.com

*Clinical and Translational Allergy* 2017, **7(Supple 4):**O01

**Background:** Methylisothiazolinone (MI) is used as a preservative in occupational, domestic products and, since 2005, in cosmetics. It is a part of the preparation of methylchloroisothiazolinone (MCI)/MI and only in the last years started to be tested as a single allergen in the baseline series. According to recent studies, the sensitization rate for MI varies between 1 and 6%, with a marked increase, particularly in the late years. Objectives: To describe patients sensitized to MI and MCI/MI and investigate its prevalence among patch tested patients

**Methods:** Cross-sectional retrospective study, including all patients who performed, from 2011 to 2016, the European and Portuguese baseline patch test series, in a Dermatology department of a tertiary hospital. Those positive to MI and/or MCI/MI, were selected.

**Results:** During the study period, 1768 patients (70% women), performed the baseline series; 972 (55%) tested positive according to the International Contact Dermatitis Research Group’s scoring system. Of these, 114 individuals (11.7%) presented positivity to MI and/or MCI/MI; 81 (71%) were women; mean age (minimum-maximum) of 44.3 (8–86) years; 33.3% had previous history of atopic comorbidities. Hands either isolated or associated with other body parts were the most frequent symptomatic location (54 cases, 47.4%) followed by generalized dermatitis (33 cases, 29%). Further comparison of MI sensitization revealed no significant differences with respect to gender (p = 0.267), age (p = 0.616), or the presence of allergic comorbidities (p = 0.536). In 2011, only one patient (0.3%) tested positive to MCI/MI and after June 2012 patients (1.1%) were positive to MI. After 2012 we observed a significant increase in MI sensitization, which rose from 5.7% in 2013 to 6.3, 11.9 and 12.2% in 2014, 2015 and 2016, respectively.

**Conclusions:** We observed that, between 2012 and 2016, sensitization to methylisothiazolinone has grown more than tenfold. These data provide further evidence of the increasing MI sensibilization epidemic over the last years, as reported in several European countries, and reinforces the need to determine safer use concentrations in the products, namely, rinse-of cosmetics.

### O02 Assessment of aggregate consumer exposure to isothiazolinones via cosmetics and detergents

#### Elena Garcia Hidalgo, Natalie Von Goetz, Konrad Hungerbühler

##### ETH Zürich, Zürich, Switzerland

###### **Correspondence:** Elena Garcia Hidalgo - gelena@student.ethz.ch

*Clinical and Translational Allergy* 2017, **7(Supple 4):**O02

**Background:** Isothiazoliones can cause allergic contact dermatitis and are present in a variety of consumer products, such as cosmetics, detergents and do-it-yourself products. Skin sensitization is induced following dermal exposure to a sensitizer in an amount exceeding the sensitization threshold. The critical determinant of exposure for evaluating skin sensitization risks is dose per unit area of exposed skin. Therefore, aggregate exposure per body site should be assessed as the basis for a quantitative risk assessment (QRA). At present, aggregate (non-food) consumer exposure is not routinely evaluated and most available computer models are not suited for estimating aggregate exposure. In the past few years a few models were developed which are able to perform probabilistic aggregate consumer exposure assessments. Nevertheless, one of the things hampering the risk assessment of sensitizing substances (including isothiazolinones) is a lack of relevant exposure data. For example, information is often not available on use amounts, frequency and duration of use and on concentrations of substances in products. The aim of this study is to estimate aggregate exposures to four isothiazolinones (Methylisothiazolinone, Chloromethyisothiazolinone, Benzisothiazolinone, and Octylisothiazolinone) that are found in many products, which are often concurrently used by the same consumer.

**Methods:** We are using a dermal exposure model requiring consumer product use and isothiazolinone’s concentration data as input variables. Product use was determined by a postal questionnaire in Switzerland, which included children and adolescents, providing for the first time in Europe information regarding detergents combined together with cosmetics. Isothiazolinone’s concentrations were measured in these products that were frequently used by the survey respondents.

**Results:** Individual-based aggregate exposure was estimated by combining the reported individual use patterns with the isothiazolinones concentrations in the products used by the individual person (which were measured in a parallel analytical study). Thus, for each substance we provide realistic distributions of exposure for both genders and across all age groups.

**Conclusions:** The exposure factors and calculation strategies developed in this study can serve as a basis for exposure assessments for other sensitizing substances.

### O03 The clinical time-course of diphenylcyclopropenone-induced contact allergy in healthy humans following repeated epicutaneous challenges

#### Kristian Fredløv Mose^1^, Flemming Andersen^1^, Lone Skov^2^, Mads Røpke^3^, Thomas Litman^3^, Peter Friedmann^4^, Klaus Ejner Andersen^1^

##### ^1^Department of Dermatology and Allergy Centre, Odense University Hospital, University of Southern Denmark, Odense, Denmark; ^2^Department of Dermato-Allergology, Herlev and Gentofte Hospital, University of Copenhagen, Hellerup, Denmark; ^3^Departments of Clinical Pharmacology and Molecular Biomedicine, LEO Pharma A/S, Ballerup, Denmark; ^4^Division of Infection, Inflammation and Immunity, Sir Henry Wellcome Laboratories, Southampton University Hospitals NHS Trust, Southampton, United Kingdom

###### **Correspondence:** Kristian Fredløv Mose - kristian.mose@rsyd.dk

*Clinical and Translational Allergy* 2017, **7(Supple 4):**O03

**Background:** The immune reactivity exhibited by newly sensitized individuals following repeated exposure to a contact allergen remains largely unknown. Recent evidence suggests that repeated challenges with a potent hapten, diphenylcyclopropenone (DPCP), could result in an initial augmentation of immune responses followed by a response plateau.

**Methods:** We set out to determine whether repeated exposure to DPCP drives the immune reactivity to ever-higher levels or results in a response plateau in newly sensitized individuals. Ten healthy volunteers were sensitized to DPCP followed by five or six elicitation challenges with DPCP at four week intervals. The responses were assessed using a visual score and quantified as skinfold thickness measured with a caliper.

**Results:** The visual grades of responses to repeated monthly challenges with DPCP reached a plateau after two challenges, while the responses measured with calipers attained the plateau after 3 challenges. The almost identical time course of contact allergy as observed by visual scores and caliper readings, respectively, generated a strong positive correlation coefficient (R^2^ = 0.9337) indicating a clear linear association between visual scores and skinfold thickness.

**Conclusions:** We have shown that in de novo sensitized individuals, repeated challenges with DPCP result in immune responses with constant levels of reactivity over time. The clinically quantifiable and reproducible responses elicited in this inflammatory model can potentially be used to compare the anti-inflammatory effects of topical immunomodulating agents, including steroids.

### O04 High dose of Omalizumab (450 mg) is beneficial for patients with severe, unresponsive chronic spontaneous urticaria—real life experience

#### Ilan Asher^1^, Keren Mahlab-Guri^1^, Shira Bezalel-Rosenberg^1^, Ben Werner^1^, Daniel Elbirt^1^, Elias Elias Toubi^2^, Zahava Vadasz^2^, Zev Sthoeger^1^

##### ^1^Kaplan Medical Center, Rechovot, Israel; ^2^Bnai-Zion Medical Center, Haifa, Israel

###### **Correspondence:** Ilan Asher - asherdn@gmail.com

*Clinical and Translational Allergy* 2017, **7(Supple 4):**O04

**Background:** Objectives: To evaluate the real life efficacy of high dose (450 mg monthly) of Omalizumab in the treatment of chronic spontaneous urticaria (CSU) patients who did not response to standard (300 mg monthly) Omalizumab dose.

**Methods:** 50 patients with severe CSU unresponsive to standard treatment (combination of high dose (X4) antihistamines, montelukast, corticosteroids and/or cyclosporine) were treated with Omalizumab (≥ 3 doses, 300 mg monthly) for a mean period of 16.3 ± 10.4 (range 3–46) months. Disease severity was defined by UAS7 score. Response was defined as complete (UAS7 ≤ 6, or improvement of > 70% in UAS7 from baseline), partial (improvement of 50–70% in UAS7) or failure (< 50% improvement). Patients who failed or partially response to 6 or more Omalizumab injections were offered higher dose (450 mg monthly).

**Results:** Our patients (68% females, mean age 48 ± 20 years) had CSU for 5.2 ± 9.3 (range 0.8–51) years. Their mean UAS7 prior to Omalizumab treatment was 30 ± 8 (range 14–42). Following Omalizumab, their UAS7 decreased to 7 ± 10 (p < 0.001).The Response rate was complete in 30 (60%), and partial in 15 (30%) patients. 5(10%) patients failed Omalizumab. The mean time to response was short (1.9 ± 2 months). 9 patients (8 partial responders, 1 failure) received higher dose of Omalizumab (450 mg monthly). Following the initiation of Omalizumab, 8 patients (6 with complete response and 2 with partial response) improved significially and only one failed the higher dose. The mean time to response was 2.6 ± 0.9 months. The mean UAS7 decreased in those patients from 20 ± 9 to 7 ± 10 (p = 0.002).

**Conclusions:** High dose (450 mg monthly) of Omalizumab is highly beneficial in CSU patients who failed (or partially response) standard (300 mg monthly) Omalizumab treatment.

### O05 Pattern of symptoms after standardized challenges with foods and drugs; data from a highly-specialized single-center study

#### Esben Eller, Charlotte Gotthard Mortz, Carsten Bindslev-Jensen

##### Odense University Hospital, Odense C, Denmark

###### **Correspondence:** Esben Eller - esben.eller@rsyd.dk

*Clinical and Translational Allergy* 2017, **7(Supple 4):**O05

**Background:** Allergic reactions manifest differently depending of age, challenge item and route of administration. Few studies have, however, addressed this in a systematic way based on challenge data using standardized end-points. We aimed to address pattern of symptoms from challenges with food and drugs, respectively, performed according to EAACI guidelines at a highly-specialized single-center study.

**Methods:** The basis for this comparison was symptoms and signs from all positive food (n = 2382) and drug (n = 428) challenges recorded at the Allergy Center, Odense University Hospital, Denmark from 2001–2016. Egg (n = 720), peanut (n = 579), hazelnut (n = 264) and milk (n = 230) were the most frequent food challenges, which due to age heterogeneity were divided into 3 age groups; 0–3Y (n = 859), 4–15Y (n = 990) and +15Y (n = 533). 2/3 of drug challenges were with antibiotics (n = 285), the remaining with NSAID (n = 143).

**Results:** Urticaria (47%) was the most frequent symptom after food challenges, followed by GI symptoms, however both were significantly affected by patient’s age. For urticaria, infants more often had urticaria (64% among 0–3Y) than older patients (34% in the +15Y group). Infants had significantly fewer GI symptoms, both subjective (OAS, abdominal pain, nausea) and objective symptoms (emesis and diarrhea), than older children and adults. Peanut more often caused rhino-conjunctivitis and/or severe objective GI symptoms (vomiting and diarrhea) compared to milk and egg. Skin symptoms were common after drug challenges (68%), i.e. either localized or generalized pruritus (47%), urticaria (36%), rash (35%) or angioedema (17%). Challenge with NSAID significantly more often caused urticaria, upper respiratory symptoms (rhino-conjunctivitis), respiratory distress and lower respiratory symptoms than challenges with antibiotics. In contrast, rash and hypotension were more often elicited by antibiotics than NSAID.

**Conclusions:** Skin symptoms are common after drug challenges but also as stop-criteria in food challenges. NSAID elicit respiratory symptoms in contrast to antibiotics. GI symptoms are common in food—but not in drug challenges.

### O06 Misdiagnosis and mistreatments of hereditary angioedema in 83 pediatric and adult patients

#### Maryam Ayazi^1^, Mohammad Reza Fazlollahi^1^, Shiva Saghafi^1^, Sajedeh Mohammadian^1^, Iraj Mohammadzadeh^2^, Mohammad Hassan Bemanian^3^, Abbas Fayazi^4^, Mohammad Nabavi^3^, Masoud Movahedi^5^, Seyed Alireza Mahdaviani^6^, Najmoddin Kalantari^5^, Maryam Mahloujirad^1^, Bahram Mir Saeed Ghazi^7^, Taher Cheraghi^8^, Javad Ghaffari^9^, Akefeh Ahmadiafshar^10^, Marzieh Heidarzadeh Arani^11^, Homa Sadri^12^, Raheleh Shokouhi Shoormasti^1^, Zahra Pourpak^1^

##### ^1^Immunology, Asthma and Allergy Research Institute, Tehran University of Medical Sciences, Tehran, Iran; ^2^Non-Communicable Pediatric Diseases Research Center, Babol University of Medical Sciences, Babol, Iran; ^3^Department of Clinical Immunology and Allergy, Hazrate Rasoul Hospital, Iran University of Medical Sciences, Tehran, Iran; ^4^Division of Allergy and Immunology, School of Medicine, Ahvaz University of Medical Sciences, Ahvaz, Iran; ^5^Department of Immunology and Allergy, Children’s Medical Center, Tehran University of Medical Sciences, Tehran, Iran; ^6^Pediatric Respiratory Diseases Research Center, National Research Institute of Tuberculosis and Lung Diseases (NRITLD), Shahid Beheshti University of Medical Sciences, Tehran, Iran; ^7^Department of Immunology, Bahrami Hospital, Tehran University of Medical Sciences, Tehran, Iran; ^8^Department of Pediatrics, 17th Shahrivar Children’s Hospital, Guilan University of Medical Sciences, Rasht, Iran; ^9^Department of Pediatrics, Mazandaran University of Medical Sciences, Sari, Iran; ^9^Department of Pediatrics, Mousavi Hospital, Zanjan University of Medical Sciences, Zanjan, Iran; ^10^Department of Pediatrics, Kashan University of Medical Sciences, Kashan, Iran; ^11^Department of Pediatrics, Shahid Bahonar Hospital, Alborz University of Medical Sciences, Karaj, Iran

###### **Correspondence:** Maryam Ayazi - marie.ayazi@yahoo.com

*Clinical and Translational Allergy* 2017, **7(Supple 4):**O06

**Background:** HAE (Hereditary Angioedema) is a disorder caused by a deficiency of C1INH (C1 Inhibitor) manifesting with subcutaneous and/or submucosal edema attacks. Owing to the rare nature of HAE, it is often misdiagnosed, in turn leading to mistreatments. In this regard poor-controlled laryngeal edema, abdominal pain mistaken for acute abdomen, and recurrent swellings result in mortality, unnecessary surgeries, and notable reduced quality of life, respectively. Hence, we to investigate the former misdiagnosis and mistreatments of referred HAE cases.

**Methods:** A through history including demographics, clinical signs, and past drug history along with informed consent was obtained from HAE suspected patients referred to Immunology, Asthma and Allergy Research Institute in 2006–2016. Patients with HAE diagnosis, made based on low levels of C1-INH and C4 with suggestive clinical findings (having ruled out other possible etiologies) enrolled into this study and registered in Iranian HAE registry.

**Results:** Among 83 patients with HAE definite diagnosis, 57% had until then received prophylactic and 74% on-demand medications, among which antihistamines (61%) and steroids (66%) were the most commonly used, respectively. As prophylaxis, FFP (fresh frozen plasma) in 1 patient, psychiatric medications (eg. alprazolam) in 3, and danazole (but with wrong dose) in 2 patients had been prescribed. NSAIDs, antispasmodics, PPIs and H2 blockers had been also used in abdominal pain episodes (Table 1). Antihistamines were ineffective for prophylaxis in 50% patients, while the majority of patients (74%) reported partial to complete symptom relief following on-demand steroids. However, on-demand steroid intake revealed no significant association with reduction of severe outcomes (abdominal surgery or tracheostomy).

**Table 1:** Results

Treatment/drugs	1st AH	2nd AH	Steroids	NSAIDs	H2 B/PPI/AntiSpa^a^	Epineph	Colchicine	Doxepin
Prophylaxis (%)	24.4	51	19.5	0	7	0	7	5
On demand (%)	28.2	41	66	7	11.6	7.1	0	0

*1st AH* first generation H1 antihistamine, *2nd AH* second generation H1 antihistamine, *H2 B* h2 blocker, *PPI* proton pump inhibitor, *AntiSpa* antispasmodic

^a^Prescribed for attacks of abdominal pain

**Conclusions:** This HAE treatment pattern suggests that it had been misdiagnosed for allergic or inflammatory disorders and accordingly treated. These medications were surprisingly found to be rather effective from the patients’ perspective, although abdominal surgeries and tracheostomy were not decreased after treatment. The spontaneous regression of the symptoms as well as placebo effect might be involved in patients’ idea of the drug effectiveness. However, questions still remain regarding involvement of other possible pathophysiological mechanisms. In conclusion, considering notable HAE mistreatment, improving awareness of health care providers may be helpful to reach standard management.

## Friday, 28 April 2017

### O07 The involvement of LELP1 gene polymorphisms in atopic dermatitis development

#### Martyna Wesserling^1^, Magdalena Trzeciak^2^, Jolanta Glen^2^, Marzena Grden^1^, Roman Nowicki^2^, Tadeusz Pawelczyk^1^

##### ^1^Department of Molecular Medicine, Medical University of Gdansk, Gdansk, Poland; ^2^Department of Dermatology, Venereology and Allergology, Medical University of Gdansk, Gdansk, Poland

###### **Correspondence:** Martyna Wesserling - martynawesserling@gmail.com

*Clinical and Translational Allergy* 2017, **7(Supple 4):**O07

**Background:** Atopic dermatitis (AD) is a common, chronic, inflammatory skin disease. It is characterized by the appearance of red inflammatory foci, itch and dry skin. Despite carrying out many studies all over the world, the pathogenesis of AD remain unrevealed in details. The research hypothesis lays on an assumption that, the development of allergic diseases including atopic dermatitis is related to an excessive penetration of allergens into the body caused by changes within the cornified envelope proteins. These changes could be the result of the presence of various polymorphic variants of genes encoding these proteins.

**Methods:** The study population consisted of 256 subjects (152 patients with atopic dermatitis and control group of 104 healthy volunteers with no allergic diseases and immune disorders). Rs4845529, rs149304390, COSM529080 gene polymorphisms were analyzed by PCR-RFLP method using the restriction enzymes. The frequency of the rs140466902, rs142724966, rs144538546 polymorphism were determined using the molecular probes. The mRNA level of *LELP1* was analyzed by the real-time PCR.

**Results:** The obtained results indicated that some polymorphic variants (rs4845529[C] and rs144538546[A]) in the region of *LELP1* gene were associated with the development of atopic dermatitis and severity of the disease. The presence of rs4845529[C] in AD patients was associated with elevated total serum IgE level. Polymorphic variant rs144538546[A] was associated with increased itching. None of analyzed polymorphisms were associated with the expression level of *LELP1* gene.

**Conclusions:** In summary, our study provides data indicating the correlation of frequency of polymorphisms in the region of LELP1 gene of patients with AD with factors that determine the clinical phenotype of this disease.

The study was supported by grant MN 01-0164/08/249 from Medical University of Gdansk.

### O08 Temperature Controlled Laminar Airflow (TLA) benefits children and adolescents with severe eczema and multisystem allergic disease—open label pilot study

#### Claudia Gore^1^, Sara Fontanella^2^, Sadia Haider^2^, Adnan Custovic^2^, Robin Brian Gore^3^

##### ^1^Imperial College Healthcare NHS Trust, London, United Kingdom; ^2^Imperial College London, London, United Kingdom; ^3^Cambridge University Hospitals NHS Foundation Trust, Cambridge, United Kingdom

###### **Correspondence:** Claudia Gore - cgore@nhs.net

*Clinical and Translational Allergy* 2017, **7(Supple 4):**O08

**Background:** Children with very severe, persistent eczema have limited treatment options, and often require systemic immunosuppression. Effective new treatments are urgently required. We aimed to evaluate the effect of the temperature-controlled laminar airflow (TLA) device in children/adolescents with severe atopic eczema and allergic multisystem disease.

**Methods:** In an open-label study, we recruited 15 children aged 2–16 years (median 10, interquartile range [IQR] 7.5–12) with longstanding severe eczema (median duration 116.5 [82–145.5] months), sensitization to ≥ 1 perennial allergen, and multiple atopic comorbidities (15/15 rhino-conjunctivitis, 14/15 food allergy, 11/15 asthma). Run-in period of 6–10 weeks (3 visits, median 7.14 weeks) was followed by a six-month treatment period using overnight TLA (Airsonett®, Sweden). Main outcome measures were SCORAD-Index and Investigator Global Assessment (0–5 scale, IGA), and secondary outcomes included child/family dermatology quality of life (CDQLI, FDQLI), POEM, medication requirements, and healthcare contacts (during the 6 months before/after TLA-start). Analysis using Wilcoxon rank signed test, with reported 2-sided significance level at p < 0.1.

**Results:** There were no significant changes during the run-in period for any of the outcome measures. We observed a significant improvement in SCORAD after the 6-month TLA-treatment period, from 34.9 [28.75–45.15] to 24.1 [18.75–37.55], p = 0.026. IGA improved from a median of 4 [3–4] to 3 [1.5–3.5], p = 0.09. Similarly, there was a significant improvement in FDQLI (16.0 [12.0–19.0] to 11 [6.5–14.5], p = 0.054). We observed no significant changes in CDQLI or POEM. Clinical improvement in eczema severity during the treatment period was accompanied by a significant reduction in the requirement for potent topical corticosteroids (p = 0.033) and number of hospital-contacts for eczema (p = 0.082).

**Conclusions:** TLA treatment leads to a significant clinical improvement in children with severe atopic eczema, with accompanying reduction in medication usage and healthcare utilization. A randomized controlled trial is urgently required.

### O09 Skin symptoms in reported drug hypersensitivity reactions in children

#### Neringa Stirbiene, Odilija Rudzeviciene

##### Vilnius University Faculty of Medicine, Children Hospital Affiliate of Vilnius University Hospital Santariskiu klinikos, Pediatrics Centre, Vilnius, Lithuania

###### **Correspondence:** Neringa Stirbiene - neri_but@yahoo.com

*Clinical and Translational Allergy* 2017, **7(Supple 4):**O09

**Background:** Patients/parents usually report skin symptoms in suspected drug hypersensitivity reactions in children. The aim of our study was to analyse the incidence and clinical pattern of skin symptoms in suspected drug hypersensitivity reactions in children who were tested for drug allergy.

**Methods:** 41 children were tested for drug allergy in Vilnius University Hospital from 2014 to 2016: 24 (58.54%) girls and 17 (41.46%) boys. The mean age of children was 7.42 ± 5.18 (3 months–17 years) years old.

**Results:** 60 drug hypersensitivity reactions were reported in our study. Four drug hypersensitivity reactions were reported in one child, three reactions – in 4 children, two reactions – in 6 children. 18 (30.0%) reactions were immediate type. Skin symptoms were reported in 55 (91.67%) suspected drug hypersensitivity reactions. Maculopapular rash was the most frequently reported (25 (41.67%) cases), followed by macular rash (14 (23.33%) cases), urticaria 16 (26.67%) cases) and angioedema (12 (20.0%) cases). Respiratory (5 (8.33%)), cardiovascular (5 (8.33%)) and gastrointestinal symptoms (3 (5.0%)) were reported less frequently. Antibiotics were the main suspected drugs (44 (73.33% reactions), followed by local anesthetics (9 (15.0%) reactions) and NSAIDs (5 (8.33%) reactions). Amoxicillin was the most frequently reported culprit drug (21 (35.0%) reactions). Four (9.76%) children were confirmed as being allergic, one patient to cefuroxime and cefotaxime, two patients to amoxicillin and one patient to ibuprofen. All these patients experienced skin symptoms in drug hypersensitivity reactions.

**Conclusions:** Maculopapular and macular rash were the most frequently reported in suspected drug hypersensitivity reactions. Antibiotics were the main suspected culprit drugs. Drug allergy was confirmed in only one tenth of children with skin symptoms in our study. Only complete allergological diagnostic work-up can confirm drug allergy.

### O10 Primary sensitization versus co-sensitization to hydrolyzed wheat protein

#### Morten J. Christensen^1^, Per Stahl Skov^2^, Lars K. Poulsen^3^, Carsten Bindslev-Jensen^1^, Charlotte G. Mortz^1^

##### ^1^Department of Dermatology and Allergy Center, Odense Research Center for Anaphylaxis, Odense, Denmark; ^2^Reflab Aps, Copenhagen, Denmark; ^3^Allergy Clinic, Copenhagen, Denmark

###### **Correspondence:** Morten J. Christensen - morten.junker.christensen@rsyd.dk

*Clinical and Translational Allergy* 2017, **7(Supple 4):**O10

**Background:** Hydrolyzed wheat proteins (HWP) are used in a variety of products ranging from cosmetics to foods. Recently, severe type I allergic reactions to HWP in foods have been reported. The aim of this study was to characterize and evaluate patients with a case-history of anaphylaxis related to a food product containing HWP and, further, to describe patients with other phenotypes of wheat allergy co-sensitized to HWP.

**Methods:** We investigated 56 patients (age 1.1–78.6 years) with a sensitization to HWP either by specific-IgE (sIgE), basophil histamine release (BaHR) and/or Skin Prick Test (SPT). Based on case-history the population was divided into three groups, 1: anaphylaxis elicited by ingestion of HWP product (n = 9), 2: wheat-induced anaphylaxis (WIA) (n = 19) and 3: wheat-dependent, exercise-induced anaphylaxis (WDEIA) (n = 28). All patients were examined with detailed case-history, SPT, sIgE and BaHR followed by an open food challenge (WDEIA ± exercise).

**Results:** In total the positive rate of sIgE to HWP was 83.9% (47/56), SPT 83.3% (35/42) and BaHR 52.3% (22/42). Group 1: Nine patients, all wheat tolerant and mono-sensitized to HWP with sIgE (median 5.3 kU/L, [2.12;21.9]), SPT (median 6.0 mm, [5.5;16.5]) and BaHR (median threshold level 0.003 µg/mL, [0;0.1]) significantly higher than group 2 and 3 (p < 0.05). Group 2: The positive rate of sIgE to wheat was 100% (19/19) and to HWP 84.2% (16/19) (median 2.1 kU/L, [0.02;56.6]). Group 3: The positive rate of sIgE to omega-5-gliadin was 82.1% (23/28) and to HWP 78.6% (22/28) (median 1.2 kU/L, [0.0;19.2]). A complete negative pattern of IgE sensitization compared to the other phenotypes of wheat allergy was found in group 1 including , omega-5 gliadin (f416), gliadin (f98), High Molecular Weight (Tri a 26) and α-amylase trypsin inhibitor (Tri a 30).

**Conclusions:** Reactivity to HWP seems to be confined to patients sensitized to this heterogeneous group of products without concomitant allergy to unmodified wheat. Irrelevant co-sensitization is also seen in classical wheat allergy. A striking finding was an ultrahigh reactivity in BaHR in patients with allergy to HWP.

### O11 Multiple drug hypersensitivity is not infrequent in combination therapy

#### Oliver Hausmann^1^, Yuttana Srinoulprasert^2^, James Yun^3^, Werner Pichler^4^

##### ^1^Loewenpraxis, Luzern, Switzerland; ^2^Department of Immunology, Faculty of Medicine Siriraj Hospital, Mahidol University, Bangkok, Thailand; ^3^Department of Clinical Immunology, Royal Prince Alfred Hospital, Sydney, Australia; ^4^ADR-AC GmbH, Bern, Switzerland

###### **Correspondence:** Oliver Hausmann - hausmann.allergie@hin.ch

*Clinical and Translational Allergy* 2017, **7(Supple 4):**O11

**Background:** Multiple drug hypersensitivity (MDH) is a syndrome that develops as a consequence of massive T cell stimulations and is characterized by long lasting drug hypersensitivity reactions (DHRs) to different drugs. Initial symptoms are mostly severe exanthems or drug rash with eosinophilia and systemic symptoms (DRESS). Subsequent symptoms due to another drug often appear in the following weeks or months to years and frequently differ in clinical presentation. The eliciting drugs can be identified by positive skin or in vitro tests.

**Methods:** We performed a retrospective analysis > 2000 LTT analyzed over 6 years (2005–2010) for the frequency of single and double sensitizations within drug allergic patients involving 4 commonly used drug combination therapies (amoxicillin/clavulanic acid, trimethoprim/sulfamethoxazole, piperacillin/tazobactam, sulfasalazine metabolites).

**Results:** LTT results of patients who had DHR to a combination therapy are shown in Table 1. For amoxicillin/clavulanic acid and trimethoprim/sulfamethoxazole severe exanthems were the main presenting symptom, whereas initial reactions to piperacillin/tazobactam and sulfasalazine metabolites where mainly DRESS. Patients with DRESS to the above mentioned combination therapy were positive in 65–85% of LTT.

**Table 1:** LTT results of patients who had DHR to a combination therapy

Total pos. LTT	Pos. LTT/%	Pos. LTT/%	Pos. LTT/%
AMX/CLA	Amoxicillin (AMX)	Clavulanic acid (CLA)	Both
n = 115	64 (56%)	12 (10%)	39 (34%)
TMP/SMX	Trimethoprim (TMP)	Sulfamethoxazole (SMX)	Both
n = 37	4 (11%)	16 (43%)	17 (46%)
PIP/TZB	Piperacillin (PIP)	Tazobactam (TZB)	Both
n = 21	7 (33%)	7 (33%)	7 (33%)
Sulfasalazine	Sulfapyridine	5-Aminosalicylic acid (5-ASA)	Both
n = 10	6 (60%)	0 (0%)	4 (40%)
Total n = 183	93 (33–60%)	23 (0–33%)	67 (33–46%)

**Conclusions:** The drugs involved in starting MDH are the same as for DRESS and are usually given in rather high doses. Fixed drug combination therapies are frequently involved in MDH and 30–40% of patients with severe DHR to combination therapy show T cell reactions to *both* components. As this is substantially higher than the reaction to the less immunogenic compound. Either the immunogenic drug or the high dose of combination therapy enhances T cell reactivity to the less immunogenic drug of the combination therapy as well.

## Poster discussion session. Topic 1: Urticaria

### P01 Omalizumab facilitates cessation of immunosuppressant in severe chronic spontaneous urticaria

#### Kok Loong Ue, Keyna Bintcliffe, Siobhan Gilkes, Clive Grattan, Krzysztof Rutkowski

##### Guy’s and St Thomas’ NHS Foundation Trust, London, United Kingdom

###### **Correspondence:** Kok Loong Ue - Kokloong.Ue@gstt.nhs.uk

*Clinical and Translational Allergy* 2017, **7(Supple 4):**P01

**Background:** Omalizumab (anti-IgE) is a new 2nd line therapy for chronic spontaneous urticaria (CSU) non-responsive to H_1_-antihistamines. It is well tolerated and no regular monitoring is required. Previously, oral corticosteroids (OCS) and immunosuppressants such as ciclosporin, mycophenolate mofetil and methotrexate were used as 2nd/3rd line treatment. They carry risks of adverse reactions. Hence, regular blood monitoring is needed, which is costly and time consuming.

**Methods:** A retrospective case review of all patients treated with 300 mg omalizumab every 4 weeks was undertaken to assess if it allowed for immunosuppressant and/or OCS withdrawal. Urticaria activity score 7 (UAS7) was used to monitor response, which was defined as a reduction (UAS7 < 16) or complete resolution of symptoms (UAS7 = 0).

**Results:** 91 patients (60 female; mean age of 44) were treated with omalizumab between November 2015 to October 2016. 93.4% (85/91) patients responded by the 5th injection, with 68.2% (58/85) responding to the 1st injection. In the responder group, at the time of initiation of omalizumab, 28 patients were on prednisolone, 5 on immunosuppressant (3 ciclosporin, 1 methotrexate, 1 mycophenolate mofetil) and 6 on both prednisolone and immunosuppressant (3 ciclosporin, 2 methotrexate, 1 mycophenolate mofetil). In these 39 patients, mean pre-omalizumab UAS7 was 36 despite treatment. 88.2% (30/34) of patients on prednisolone successfully stopped it and 11.8% (4/34) were able to taper the dose but not fully withdraw due to underlying steroid-induced adrenal insufficiency. 90.9% (10/11) patients on immunosuppressant successfully discontinued them and 9.1% (1/11) remained on it for non-CSU reason (atopic dermatitis). Interestingly, all but 2 of these 39 patients had a negative basophil histamine release assay (BHRA) with a poor response to immunosuppressant before starting omalizumab. Moreover, in the group of 85 responders, 14 more patients had been on prednisolone and 39 more had failed to respond to one or more immunosuppressants at some point before the initiation of omalizumab.

**Conclusions:** Omalizumab facilitates cessation of immunosuppressant in CSU patients. It has a good safety profile and maintains its efficacy during repeated courses. It is a reliable treatment option, which obviates the need for long-term OCS or immunosuppressant in most patients. Moreover, these results support our previous finding that a positive BHRA is a marker for immunosuppressant-responsive CSU.

### P02 Autoantibody quantity and affinity analysis in chronic autoimmune urticaria patients

#### Nicole Wirth^1^, Tatjana Pecaric-Petkovic^1^, Antonia Bünter^1^, Werner Pichler^1^, Oliver Hausmann^2^

##### ^1^ADR-AC GmbH, Bern, Switzerland; ^2^ADR-AC GmbH/Löwenpraxis, Bern/Lucern, Switzerland

###### **Correspondence:** Nicole Wirth - nicole.wirth@adr-ac.ch

*Clinical and Translational Allergy* 2017, **7(Supple 4):**P02

**Background:** Ca. 35–50% of patients with chronic urticaria (CU) have functional IgG autoantibodies (autoAbs) against the α-subunit of the high affinity IgE receptor (anti-FcεRIα) and/or against mast cell and basophil surface bound IgE (anti-IgE). These presumably autoimmune forms of CU can be identified by functional tests like autologous serum skin tests (ASST) or by CU-basophil activation tests (CU-BAT), which, however, are both cumbersome and not always reliable. We aim to improve and complement the CU-BAT on a blood donor free basis. Thus, we investigate the characteristics of anti-FcεRIα and anti-IgE autoAbs in CU measuring their quantity as well as their affinity and compare it to the CU-BAT.

**Methods:** We established a chaotropic ELISA measuring autoAb titer combined with antibody affinity. In a first step, autoAbs of CU-patients and control sera were quantified using a human anti-FcεRIα ELISA and human anti-IgE ELISA. In a second step, autoAb quantified serum samples were normalized to a titer in the standard range. Diluted serum samples were incubated in presence and absence of the chaotropic agent ammonium thiocyanate, which is able to disturb intermolecular forces such as antibody-antigen binding sites. Antibody affinity was determined as the percentage of still bound autoAbs quantity after ammonium thiocyanate addition.

**Results:** Anti-FcεRIα as well as anti-IgE autoAbs, which are found in CU-patient sera, differ greatly in their quantity and quality. Hence, based on autoAbs quantity and quality CU-patients can be divided in different subgroups: anti-FcεRIα respectively anti-IgE low affinity/low quantity, high affinity/low quantity, low affinity/high quantity, and high affinity/high quantity.

**Conclusions:** Subdivision in affinity/quantity groups showed a better correlation to CU-BAT patient data compared to quantity analysis alone. Determination of quantity and affinity may be helpful in determining functionally relevant autoAbs and thus substitute CU-BAT and ASST as diagnostic tests. However, the value of affinity determination still needs to be analyzed in larger patient cohorts and to be correlated to the severity of CU. As a simple ELISA test the presented CU-serum tests are promising to follow the highly variable course of CU and to monitor the response and persistence of therapeutic interventions such as anti-IgE therapy.

### P03 Urticaria Control Test (UCT)—responsiveness and minimal important difference

#### Tatevik Ohanyan^1^, Nicole Schoepke^1^, Martin Metz^1^, Thomasz Hawro^1^, Torsten Zuberbier^1^, Marcus Maurer^1^, Karsten Weller^1^, Adriane Peveling-Oberhag^2^, Petra Staubach-Renz^2^

##### ^1^Charité Universitätsmedizin Berlin, Berlin, Germany; ^2^Universitätsklinikum Mainz, Mainz, Germany

###### **Correspondence:** Tatevik Ohanyan - tatev_ohanyan@yahoo.com

*Clinical and Translational Allergy* 2017, **7(Supple 4):**P03

**Background:** The Urticaria Control Test (UCT) is a globally used and universal patient-reported outcome measure for measuring disease control in chronic urticaria patients. As of yet, its responsiveness has not been established. The aim of this study was to investigate the UCT’s ability to detect changes over time, including the minimal important difference (MID) and the smallest detectable change (SDC).

**Methods:** Sixty-five antihistamine-refractory CSU patients used the UCT to document their disease control as well as several anchor instruments for disease activity, disease control, health-related quality of life, and treatment response before and 4 weeks after the initiation of omalizumab therapy. The UCT’s sensitivity to change was assessed by correlating its score changes with changes in the applied anchors. In addition, the MID and SDC were calculated by using distribution-criterion and anchor-based approaches.

**Results:** After the initiation of omalizumab, UCT scores markedly improved as compared to pretreatment levels. The UCT score changes correlated strongly with changes of disease activity and health-related quality of life. In addition, UCT results and their changes were well in accordance with the patient’s assessment of their treatment efficacy, their disease control, and with the patient’s response to treatment. The MID and SDC of the UCT were found to be 3 and 4 points, respectively (Table 1).

**Table 1:** Results

Number of patients	65
Age in years, mean ± SD (range)	44.6 ± 14.9 (22–74)
Gender ratio; female:male	3.3 (77%:23%)
Disease duration in months, mean ± SD (range)	73.1 ± 97.0 (3–480)
Symptom pattern	
Wheals without angioedema	12 patients (18%)
Wheals and angioedema	53 patients (82%)
Angioedema without wheals	0 patients (0%)
Disease pattern	
Only CSU	29 patients (45%)
CSU plus CIndU*	32 patients (49%)
Unknown if CIndU in addition to CSU	4 patients (6%)

**Conclusions:** The UCT score is sensitive to change. Accordingly, the UCT is a valuable tool to assess levels but also changes of disease control in patients with chronic urticaria over time, e.g. before and after treatment adjustment.

### P04 Effect of histamine-free diet in adult patients with chronic urticaria

#### Yong Won Choi^1^, Jee Hee Son^2^, Yong Se Cho^2^, Bo Young Chung^2^, Hye One Kim^2^, Chun Wook Park^2^

##### ^1^Kangnam Sacred Heart Hospital, Seoul, South Korea; ^2^Department of Dermatology, Kangnam Sacred Heart Hospital, Seoul, South Korea

###### **Correspondence:** Yong Won Choi - henric@naver.com

*Clinical and Translational Allergy* 2017, **7(Supple 4):**P04

**Background:** In adult patients with chronic urticaria (CU), causal relationship between food and disease exacerbation is relatively weak compared to childhood patients. However, there are many patients who report food-related aggravation of CU, and some of them may have histamine intolerance. The aim of this study was to evaluate the role of ingested histamine and to investigate the effect of histamine-free diet in adult patients with CU. port food-related aggravation of CU, and some of them may have histamine intolerance.

**Methods:** Twenty-two adult patients with CU were enrolled. Foods with high amounts of histamine were prohibited to all patients for 4 weeks. Department of nutrition supplied the reference menu with histamine-free diet and consulted the patients. Severity degree of urticaria using Urticaria Severity Score (USS) and Urticaria Activity Score (UAS) were evaluated. Plasma histamine levels and diamine oxidase (DAO) activity were determined and compared between baseline and after the histamine-free diet.

**Results:** Twenty-two adult patients were recruited and completed the 4 weeks of histamine-free diet. There was a significant difference in plasma histamine level between baseline and after the histamine free-diet (*p* = 0.014). DAO activity did not change after the histamine-free diet (*p* = 0.165). Likewise, both USS and UAS score significantly decreased after the histamine-free diet (*p* = 0.010, *p* = 0.006).

**Conclusions:** Ingested histamine might be related with CU severity and histamine-free diet is helpful for adult patients with CU. Baseline and after the histamine free-diet (*p* = 0.014). DAO activity did not change after the histamine-free diet (*p* = 0.165). Likewise, both USS and UAS score significantly decreased after the histamine-free diet (*p* = 0.010, *p* = 0.006).

### P05 Chronic urticaria—overview of 6 years

#### Isabel Rosmaninho, Miguel Vieira, Ana Moreira, Arminda Guilherme, Jose Ferreira, Ines Lopes, Jose Moreira Silva

##### CHNVGaia/Espinho, EPE, Vila Nova Gaia, Portugal

###### **Correspondence:** Isabel Rosmaninho - irosmaninho@yahoo.com

*Clinical and Translational Allergy* 2017, **7(Supple 4):**P05

**Background:** Chronic urticaria (CU) may occur as chronic spontaneous urticaria (CSU) or inducible chronic urticaria (CIndUs) and they may coexist. It is a frequent and debilitating disease. First line treatment includes second-generation H1 anti-histamines (AH), however in some patients it is necessary to associate anti-inflammatory or biologic agents.

**Methods:** To review the clinical features, diagnosis, management and follow-up in a group of patients with CU followed in an Allergy Department. Patients with CU between 2010 and 2016 were included retrospectively. The data collected were: age, gender, disease evolution, comorbidities, etiologic diagnosis, management and follow-up.

**Results:** A total of 281 patients (222 female), mean age 41 (± 15) years, with atopy in 32.7% were studied. Angioedema was present in 26.3% of patients and 54.8% had generalized urticaria. The median time of disease evolution was 3 years (6 months–32 years). Comorbidities associated were: depression/anxiety (26.7%), allergic rhinitis (18.9%), allergic asthma (9.6%), contact dermatitis (13.5%), thyroid disease (12.8%). Regarding the etiologic diagnosis: CSU (43.1%) and CIndUs (symptomatic dermographism: 43.1%, cholinergic urticaria: 14.6%, cold urticaria: 10%, delayed pressure urticaria: 5.7%, solar urticaria: 1.1%, aquagenic urticaria: 0.4%); CSU associated with CIndUs: 8.9%. Anxiety/depression was associated with CSU (p = 0.001). All patients underwent lifestyle modification and AH treatment (on demand: 10.3%, standard dosage: 55.9%, up to 4 times: 29.2% and association with omalizumab: 4.6%. During the follow up period: 68.7% improved, 16.7% did not improve and spontaneous remission occurred in 14.6%.

**Conclusions:** The sex and mean age of the patients studied were similar to those described in literature. The most prevalent urticaria were CSU and symptomatic dermographism. Most of the patients were controlled with lifestyle modification and AH therapy. CSU was associated with a number of comorbidities. These need to be considered in the diagnosis and management of patients with CSU because conditions like anxiety/depression may have further impact on quality of life.

### P06 Cutaneous lupus as a differential diagnosis in chronic urticaria: a case report

#### Nathalie Depreux, Clara Padró, Sira Miquel, Yanina Jurgens, Albert Roger

##### Hospital Germans Trias i Pujol, Badalona, Spain

###### **Correspondence:** Nathalie Depreux - nathalie.depreux@hotmail.es

*Clinical and Translational Allergy* 2017, **7(Supple 4):**P06

Withdrawn

### P07 Face swelling: angioedema or wells syndrome? A case report

#### Alessandra Arcolaci, Nicola Vaccargiu, Margherita Deidda, Davide Firinu, Stefano Del Giacco

##### Department of Medical Sciences Clinical Allergology and Immunology University of Cagliari, Cagliari, Italy

###### **Correspondence:** Alessandra Arcolaci - alessandra.arcolaci@gmail.com

*Clinical and Translational Allergy* 2017, **7(Supple 4):**P07

**Background:** Wells syndrome (WS), also called eosinophilic cellulitis, is a rare disease, characterized by infiltrated plaques that may initially present as urticaria or cellulitis, tissue eosinophilia and blood eosinophilia (15–67%). The etiology of WS remains uncertain, have suggested possible associations with triggering factors and reactive forms to oncological, autoimmune and infectious disorders.

**Methods:** Female, 59 y-o, Hashimoto Thyroiditis. From about 1 year history of face and neck angioedema associated with recurrent herpes labialis and abnormal reaction to insect bite. Allergy and immunological screen negative. Due to the persisting of a swelling in the right submandibular region and increased inflammation markers, he admitted. ESR 91 mm, CRP 6.4 mg/l, WBC 6.3 × 10^3^/μl, EOS 0.3 × 10^3^/μl-5%, LDH 501 UI/l, IgG 2.624 mg/dl, no monoclonal gammopathy, IgEtot 4.124 KU/l, C3, C4 e C1INH normal, ANA 1:160, ENA negative. Papular itchy lesions on the extremities, submandibular lymphadenopathy, no systemic symptoms. The cutaneous biopsy showed diffuse eosinophilic infiltrate with flame figures in the dermis and subcutaneous tissue, suggestive of WS. On neck US multiple enlarged matting nodes without hilum in cervical and submandibular region. The neck-chest-abdomen CT scan confirmed a generalized lymphadenopathy and the node biopsy was suggestive for chronic lymphocytic leukemia. After the discharge, the patient showed a reactivation of herpes labialis associated with swelling of the lower lip till the submandibular right region that, due to the WS diagnosis, can be considered like an abnormal eosinophilic response to an infectious agent rather than a manifestation of acquired angioedema. In fact it solved with an adequate antiviral therapy.

**Results:** In case of WS, due to the possible association with severe diseases like lymphoproliferative disorders, a thorough clinical evaluation becomes mandatory. Some authors believe that skin lesions may be a local hypersensitivity reaction triggered by a number of causative agents like viral infections or insect bites. In case of hematologic malignancy herpes viruses can reactivate with severe complications rather than abnormal local edema. The treatment is the one of the underlying disease.

**Conclusions:** The correlation between clinical and histopathologic findings permitted to achieve the diagnosis of WS associated to a lymphoproliferative disorders. WS, due to the polymorphic clinical presentation and relative rarity, is a diagnostic challenge for the clinician.

**Consent to publish** Written informed consent was obtained from the patient involved in this study.

### P08 Practice comparison study: management of chronic spontaneous urticaria

#### Ekaterina Minkina^1^, Ekaterina Burova^2^, Michael Rudenko^3^

##### ^1^I.M. Sechenov First Moscow State Medical University Russia, Moscow, Russia; ^2^Bedford Hospital NHS, Bedford, United Kingdom; ^3^London Allergy and Immunology Centre, London, United Kingdom

###### **Correspondence:** Michael Rudenko - rudenko_michael@yahoo.com

*Clinical and Translational Allergy* 2017, **7(Supple 4):**P08

**Background:** The prevalence of CSU in the population ranges from 0.5 to 1% and the duration of the disease is generally 1–5 years, but is likely to be longer in more severe cases (1). While the pathogenesis of CSU has been extensively investigated, no theory has been conclusively proven, and a combination of mechanisms may play a role. Vasoactive mediators released from mast cells and basophils play a key role in the pathogenesis of CSU. Histamine acts on H1 receptors (85%) and H2 receptors (15%) in the skin (2).

**Methods:** We compared treatment outcomes of twenty randomly selected adult patients with СSU in Russia and in the UK. Treatment was done according to the local guidelines. Treatment with licensed doses of non-sedating H_1_ - antihistamines relieves symptoms effectively in < 50% of patients. Although guideline-recommended up-dosing up to fourfold increases symptom control in many patients, a substantial number of patients have only little benefit from H_1_ - antihistamines (1).

**Results:** In Russia Levocetirizine was used and only 25% of patients showed significant improvement with a dose of 5 mg. In the remaining group of the patients the dose was increased, but unfortunately 40% of patients achieved insufficient control of their symptoms. In the UK on the licensed dose of Rupatadine 10 mg full control was achieved in 30% of cases, but 15% of patients after up-dosing and addition of Montelukast 10 mg in line with NICE guidelines had UAS7 of more than 28, and were enrolled on anti-IgE treatment.

**Conclusions:** A 25% difference in the number of patients who achieved acceptable control of symptoms with H1 antihistamines in Russia vs UK might be explained by biases in the severity of cases referred to clinics, brand of antihistamine used as first line treatment based on local polices and possibly geographical factors.

**References**

1. Maurer M, et al. Allergy 2011;66(3):317–30.
2. Labrador-Horrillo M, et al, Drug Des Dev Ther. 2015;9: 4909–15.

## Poster discussion session. Topic 2: Atopic dermatitis

### P09 Netherton syndrome

#### Kaja Julge^1^, Terje Kukk^2^

##### ^1^Children’s Clinic of Tartu University Hospital, Tartu, Estonia; ^2^Dermatology Clinic of Tartu University Hospital, Tartu, Estonia

###### **Correspondence:** Kaja Julge - kaja.julge@kliinikum.ee

*Clinical and Translational Allergy* 2017, **7(Supple 4):**P09

**Background:** Netherton syndrome is rare autosomal recessive hereditary ichthyosiform disease. The classical triad of clinical features includes ichthyosis, hair shaft abnormalities and atopic diathesis.

**Methods:** The authors present the case of a 14-years old girl with the Netherton syndrome (molecular genetic diagnosis SPINK5 c.1048C > T p.R350X and c.2098G > T p.G700X). She has ichthyosis, bamboo hair (*trichorrhexis invaginata*), atopy: multi-sensitized, allergic rhinitis, asthma and has had once anaphylaxis due to eating fish.

She was born from the 3rd pregnancy/3rd delivery at 37w + 5d (3100 g, 50 cm). Her 4 years older brother is healthy but her sister had ichthyosiform erythrodermia and died in 1994 at two months of age due to sepsis. Our patient had in newborn period oedematous skin, erythrodermia, problems with thermoregulation, dehyratation, metabolic acidosis, bacterial infection and she was living in the couveuse with high temperature and humidity for the first weeks of life.

**Results:** She has growing slowly, her weight is 43 kg (3–10 percentile) and height 153 (3–10 percentile).She has been active, communicative and has had good results in the studies. Her skin disease has been more or less under control, she has severe allergy against fish, house dust mitmes and pollens. Her asthma is well controlled, her spirometry (FEV 1–119%) and FeNO (7–17 ppb) results are very good. She has had low level of B lymphocytes (CD19+) 3.7% (14–44), 101 (200–2100) and extremely high values of IgE, maximal value at 4 years 11532 kU/L, at 14 years 4732 kU/L. She is sensitised against many foods, house dust mitmes, epidermal allergens, pollens. The highest IgE antibodies levels: *D. pteronyssinus* 649 kU/L, *D. farinae* 517 kU/L, mix of fishes 224 kU/L, birch 262 kU/L, mix of grasses 92 kU/L, rye flour 123 kU/L, oat flour 125 kU/L. Component resolved diagnostics has been done. Very high value of IgE against paralbumin (38 ISU-E) is the reason why she is extremely sensitive against all fishes. She has always available adrenaline autoinjector. High level of IgE against arginine kinase (11 ISU-E) is related to the sensitivity against shrimps and other crustaceans. The IgE level against timothy allegen Phl p2 is 123 ISU-E and IgE against birch allergen Bet v1 is 60 ISU-E. Therefore spring and summer months are the seasons full of suffering and she needs antihistamines almost every day. The list of products she can eat is not long.

**Conclusions:** It is possible to have active life with severe disease.

**Consent to publish** Written informed consent was obtained from the guardians of the patient involved in this study.

### P10 The efficacy of allergen specific immunotherapy in atopic dermatitis patients

#### Sonila Dauti^1^, Esmeralda Shehu^2^

##### ^1^Hospital of Kavaja, Kavaje, Albania; ^2^Regional Hospital of Durres, Durres, Albania

###### **Correspondence:** Sonila Dauti - sonila_dauti@yahoo.com

*Clinical and Translational Allergy* 2017, **7(Supple 4):**P10

**Background:** Atopic dermatitis (AD) is a chronic inflammatory skin disorder resulting from the interactions of: genetic factors, immune dysfunction, exposure to allergens and infectious agents and deficiencies in skin barrier function. Allergen specific immunotherapy (AIT) has been the only relevant treatment of IgE - mediated allergic diseases resulting in an immune deviation. But its use in AD treatment is still debatable. We aimed to evaluate the clinical efficacy of AIT in AD patients sensitized to aeroallergens.

**Methods:** We included in this study 26 patients from January 2015, suffering from moderate to severe AD, accompanied by allergic upper respiratory symptoms. This group of patients was allergic to house dust mites or grass pollen allergens. They were all treated with subcutaneous AIT with conventional schemes.

**Results:** After 2 years of treatment with AIT we observed a notable clinical improvement of their dermatitis. Moreover we found a significant reduction of SCORAD index, 50% reduction in SCORAD at the end of first year and, 65% reduction at the end of the second year. A notable improvement, defined as a SCORAD reduction of > 67.6%, was observed in severe atopic dermatitis patients and 56% in moderate AD patients respectively. In 5 patients SCORAD values improved slightly. Also, rhinitis symptoms and their quality of life improved significantly.

**Conclusions:** Our study confirmed that AIT in moderate to severe AD patients sensitized to aeroallergens can be a safe alternative treatment of AD.

### P11 Specific immunotherapy in an adult with severe atopic dermatitis: case report

#### Simona Kasinskaite, Brigita Sitkauskiene

##### Department of Immunology and Allergology, Lithuanian University of Health Sciences, Kaunas, Lithuania

###### **Correspondence:** Simona Kasinskaite - simonaityte@gmail.com

*Clinical and Translational Allergy* 2017, **7(Supple 4):**P11

**Background:** Atopic dermatitis (AD) - is a common chronic inflammatory disease of the skin with multifactorial pathogenesis. It is considered that pathogenesis involves damage of the skin barrier which could be followed by an allergen sensitization via transepidermal penetration and lead to the enhanced Th2 response. Allergen specific immunotherapy (ASIT) is the only pathogenetically relevant treatment of IgE-mediated diseases. However, the use of ASIT in patients with atopic dermatitis remains controversial.

**Methods:** A 22 year-old woman attended to our department in October 2016 with a severe flare-up of atopic dermatitis. Her skin was very dry, with an erythematous maculopapular rash, plaques and lichenification on her upper extremities, trunk, neck and face. SCORAD index was estimated of 74 score. This worsening of her skin condition was continuing for a 2 year period, even following treatment consisting of emollients, topical glucocorticosteroids and oral antihistamines. Her relevant history included symptoms of atopic dermatitis from her childhood accompanied by the allergy to egg white. At that point, her skin problems were controlled only with emollients and diet, sometimes including topical glucocorticosteroids. Later, atopic dermatitis was in remission from 4 until 20 years old. During this period allergic rhinitis was diagnosed at the age of 13 due to sensitization to house dust mites. She had mild episodic symptoms of watery rhinitis, which was treated with oral antihistamines. At the age of 20 pruritic skin rash appeared all over her body, mainly on the trunk, arms, neck and face. After 2 years of treatment without any good effect, she came to our clinic for a consultation. The workup showed a normal total serum immunoglobulin E concentration, skin prick test was positive to house dust mites. Because she was highly sensitized to house dust mites, a decision was made to use subcutaneous specific immunotherapy with house dust mite allergoids using standard scheme.

**Results:** From the second month of ASIT, the patient reported an obvious improvement on her symptoms, reducing the necessity of topical corticosteroids and oral antihistamines. SCORAD index was estimated of 28 score.

**Conclusions:** This clinical case shows significant efficacy of allergen specific immunotherapy for the treatment of our patient with AD. The effectiveness of ASIT for severe atopic dermatitis is encouraging, especially in the cases when severe atopic dermatitis do not properly respond to conventional treatment.

**Consent to publish** Written informed consent was obtained from the patient involved in this study.

### P12 Mycosis fungoides—the importance of a prolonged follow-up

#### Ana Castro Neves, Ana Moreira, Isabel Rosmaninho, José Moreira Da Silva

##### Centro Hospitalar de Vila Nova de Gaia/Espinho, Vila Nova De Gaia, Portugal

###### **Correspondence:** Ana Castro Neves - ananeves6@hotmail.com

*Clinical and Translational Allergy* 2017, **7(Supple 4):**P12

Withdrawn

### P13 Interpretation of ImmunoCap ISAC results in 7 years old girl with severe refractory atopic dermatitis

#### Nino Mchedlishvili, Tamar Abramidze, Maia Gotua

##### Center of Allergy and Immunology, Tbilisi, Georgia

###### **Correspondence:** Nino Mchedlishvili - ninomchedlishvili@yahoo.com

*Clinical and Translational Allergy* 2017, **7(Supple 4):**P13

**Background:** Component resolved diagnosis (CRD), such as ImmunoCap ISAC technique, allow the determination of serum levels of IgE directed against specific allergen components and, as a result, a more detailed evaluation of IgE responses in complex cases of patients, who experience severe or atypical symptoms. The Correct interpretation of the results of CRD is essential for proper management of patients. The potential role of CRD in circumstances such as identification of culprit food allergens in case of polysensitization, as well as evaluation of the necessity of allergen immunotherapy, etc was assessed.

**Methods:** Case presentation of severe refractory atopic dermatitis.

**Results:** A 7 years old girl was admitted with a 5-years history of severe Atopic Dermatitis. She also suffered from rhinitis and recurrent wheezing associated with pollination season. Despite treatment severe refractory atopic dermatitis was presented. There was an episode of severe acute urticaria after salmon consumption. Due to many episodes of self-reported food allergy, patient was restricted in consumption of many products including milk, egg, fish, etc. Presence of pets at home from the first year of life was reported by patient’s parents during the collection of information for medical history. In order to reveal the main allergens of possible poly-sensitization and cross-reactivity for this particular case the ImmunoCAP ISAC component test was used.

**Conclusions:** There are several conclusions based on the result of ImmunoCap ISAC. The results of the test revealed the hypersensitivity to Kiwi and Cod. High levels of sIgE to fish parvalbumin, a major fish allergen and a marker of cross-reactions between different species of fish, and anaphylactic reaction (acute urticaria) of patient to salmon suggest that in this case strict avoidance of fish is recommended. This patient should carry adrenaline auto-injector for prevention of anaphylactic complications. Very high levels of sIgE to rFel d 4 Lipocalin suggest, that the patient should avoid contact with furry animals (Table 1). Patient changed the house, with no presence of cat and other pets and her skin condition significantly improved. Dermatitis was relieved, quality of life improved. The consumption of dairy products was successfully started without complications. Due to clinical symptoms and high levels of sIgE to Ragweed and Timothy grass specific immunotherapy is recommended.

**Table 1:** Results

Allergen source	Allergen component	Level of specific IgE (ISU-E)
Cod	rGad c1 parvalbumin	11
Kiwi	nAct d1 Cysteine protease	3.1
Timothy grass	rPhl p 1 Grass group 1	14
	rPhl p 5 Grass group 5	15
	rPhl p 6 Grass group 6	1.8
Japanese cedar	nCry j 1 Pectate lyase	3.9
Ragweed	nAmb a 1 Pectate lyase	37
Dog	rCan f 1 Lipocalin	6
	rCan f 2 Lipocalin	4
Horse	rEqu c 1 Lipocalin	12
Cat	rFel d 1 Uteroglobin	5.4
	rFel d 4 Lipocalin	15
Mouse	nMus m 1 Lipocalin	2.3
Cross-reactive components		
Profilin		
Birch	rBet v 2 Profilin	3.7
Latex	rHev b 8 Profilin	5
Annual mercury	rMer a 1 Profilin	2.7

**Consent to publish** Written informed consent was obtained from the guardians of the patient involved in this study.

## Poster discussion session. Topic 3: Contact dermatitis - Group A

### P14 Systemic allergic contact dermatitis from nickel still exists in Europe!

#### Marléne Anne Inger Isaksson, Maria Nilsson

##### Department of Occupational and Environmental Dermatology, Skane University Hospital, Malmö, Sweden

###### **Correspondence:** Marléne Anne Inger Isaksson - marlene.isaksson@med.lu.se

*Clinical and Translational Allergy* 2017, **7(Supple 4):**P14

**Background:** Systemic allergic contact dermatitis is a condition in which a systemic administration of a hapten in persons with contact sensitivity to the hapten leads to either clinically characteristic features or to a rash clinically indistinguishable from other types of contact dermatitis. Flare-up of previous dermatitis, dermatitis on previously unaffected skin, flexural dermatitis, or the Baboon syndrome may be seen. Three cases are presented. (1) A 20-year-old non-atopic male with a 4-month history of recurrent and pruritic eczematous lesions on arms, abdomen, and back. The patient had just returned from a trip to Asia when skin lesions appeared. Despite topical treatment with a potent corticosteroid and internal corticosteroids the eczema waxed and waned. He was eventually sent to us for patch testing. (2) A 22-year-old atopic female with a history of atopic eczema since childhood. Six months prior to presentation eczema started next to the umbilicus. Since then flares of pruritic eczema on arms, legs, and back. Despite topical corticosteroids the eczema continued. (3) A 15-year-old non-atopic female with a 6-month history of eczema next to the umbilicus, in the neck, and on the left shoulder.

**Methods:** Patch testing was carried out with the Swedish baseline series (based on the European baseline series but supplemented with other sensitizers) and an extended baseline series. Patch tests were removed after 48 hrs. and readings were performed on day (D)4 and D7.

**Results:** Strong positive reactions (+++) were observed to nickel 5.0% pet. in all 3 cases. All 3 had worn metal belt buckles next to the umbilicus. All belts had been purchased in Sweden. All buckles were positive when tested to the dimethylglyoxime test. No relapse was seen after the patients ceased to use the culprit metallic objects.

**Conclusions:** The release of nickel ions from metallic objects in close skin contact is regulated in the EU. If an object leaches nickel ions and an eczema develops at the skin site of exposure to this object, a systemic exposure from the absorption of nickel ions in the area of the dermatitis is possible and this is thought to explain the clinical picture. Avoidance of prolonged skin contact with the nickel-releasing alloys will then result in clearance of the systemic dermatitis as seen in these 3 cases.

**Consent to publish** Written informed consent was obtained from the patients involved in this study.

### P15 Patch testing update: the latest changes in the European baselines series

#### Jose Luis García-Abujeta^1^, Leticia De Las Vecillas Sánchez^2^, Mónica Antón Gironés^3^, Carlos Hernando De Larramendi Martínez^1^, Javier Montoro Lacomba^4^, Sandra Vicario García^1^, Fernando Rodríguez Fernández^2^

##### ^1^Hospital Marina Baixa, Villajoyosa, Spain; ^2^Hospital Universitario Marqués de Valdecilla, Santander, Spain; ^3^Hospital Universitario del Vinalopó, Elche, Spain; ^4^Hospital Arnau de Vilanova, Valencia, Spain

###### **Correspondence:** Jose Luis García-Abujeta - jlgabujeta@coma.es

*Clinical and Translational Allergy* 2017, **7(Supple 4):**P15

**Background:** Baselines Series (BS) of patch tests are the main tools used to diagnose patients with contact dermatitis (CD). While there is a standardized EU BS, many countries use their own specific BS. Most of the contact allergens are common in the European and the country-specific BS but there are differences in their concentrations and haptens that are exclusive in some of them. Evolution of BS is guaranteed by the continuous introduction if new substances and studies on CD show the rise and fall of allergens. In 2015 we studied the differences between the available EU BS. The purpose of this work is to evaluate the changes in the EU BS for the last two years.

**Methods:** Comparison of EU and national European BS published in 2015 with the 2017 version: Belgian (BE), British (BR), Finnish (FI), German (GE), Hungarian (HU), Italian (IT), Polish (POL), Portuguese (POR), Spanish (SP) and Swedish (SW).

**Results:** In this period of time there were no changes in the 30 haptens of the EU BS. Eighty-five haptens are involved in the different EU BS studied. The media of haptens in the EU countries BS was 30.8 (24 GE-41 BR) in 2015 and 32.3 (29 IT-41 BR) in 2017. Half of the national BS (BE, GE, IT, POR, SP) have been modified. Nine allergens are still in all of the BS in 2017 (Formaldehyde, Methylisothiazolinone-Methylchloroisothiazolinone (MI-MCI), Fragrance mix I, Peru Balsam, Colophonium, Cobalt chloride, Nickel sulfate, Potassium dichromate and Thiuram mix) (10 in 2015). Twenty-nine haptens (8 news) are exclusive to a specific-country BS: Thiourea mix (FI), Hydroperoxides of Limonene and Linalool (BE), Ylang-Ylang and Sandalwood oils (GE)… Mercury and Thiomersal are now present only in HU BS. MI is present in all European BS except in FI, HU and POR. Other emergent haptens found are: Sorbitan sesquioleate (GE, IT) and Textile dye mix (BE, IT, SW). Most of the changes of concentrations are related to preservatives: Formaldehyde (2) and MI-MCI (4).

**Conclusions:** A trend to increase the number of haptens in the European SB is observed. National differences are high, with a smaller core of common haptens Our results show that BS are in continuous movement with the appearance of new epidemic, emergent and exotic haptens and the loss of “old friends” like mercury or thiomersal. Multicentre European studies carried out to improve the efficacy of patch testing in relation with higher (or lower) concentrations of allergens need a long period of time to be assimilated in the different BS.

### P16 Successful treatment of normocomplementemic urticarial vasculitis with IL-1 receptor antagonist Anakinra: a case report

#### Lorenzo Stefano Pelloni, Giovanni Gaspare Ferrari

##### Allergology and Clinical Immunology Unit of Dermatology Department of Ente Ospedaliero Cantonale, Regional Hospital of Bellinzona e Valli, Bellinzona, Bellinzona, Switzerland

###### **Correspondence:** Lorenzo Stefano Pelloni - lorenzo.pelloni@eoc.ch

*Clinical and Translational Allergy* 2017, **7(Supple 4):**P16

**Background:** Urticarial vasculitis is considered a rare clinical pathologic entity characterized by recurrent episodes of urticaria with the histopathological features of a leukocytoclastic vasculitis of the small vessels. The variability in the clinical presentation may be due to the presence or absence of hypocomplementemia. We describe a case of normocomplementemic urticarial vasculitis refractory to conventional therapies that was successfully treated with IL-1 receptor antagonist Anakinra.

**Case report:** A 49-year-old male patient, known for intrinsic asthma under control with Montelukast and inhaled corticosteroids, showed up in our consultation presenting since 2 weeks purpuric urticarial plaques at the lower and upper limbs and angioedema of the face. General practitioner already treated him with systemic antihistamines and prednisone. We stopped systemic corticosteroids and performed a skin biopsy with direct immunofluorescence. Laboratory tests showed physiological values for complements C1, C3 and C4, negative autoantibodies (cryogobulins, ANA, ENA, ANCA) and an elevated serum erythrocyte sedimentation rate (ESR) and C-reactive protein (CRP). A total body CT scan excluded neoplastic aetiology of urticaria. Waiting biopsy results we restarted and increased the dosage of systemic corticosteroids and antihistamines without any improvement. Histological results were suggestive for an urticarial vasculitis so started a systemic treatment with Dapsone 50 mg daily, rapidly stopped because of appearance of an haemolytic anaemia and increased CO-Haemoglobin. We tried a subcutaneous therapy with Omalizumab 300 mg every 4 weeks, but after 2 injections we didn’t find any improvement. Finally we introduced Anakinra 100 mg daily, thanks to which we remarked a slowly improvement of skin lesions. After 6 weeks patient symptoms were in remission and he now benefits of this treatment since two years: he’s still in remission and without any adverse effects.

**Conclusions:** IL-1 receptor antagonist Anakinra represents an effective treatment option for normocomplementemic urticarial vasculitis refractory to the conventional therapies or to Omalizumab. Treatment is safe and well tolerated by the patient and leads to complete and sustained remission within 4 to 6 weeks without relapse in the most cases.

**Consent to publish** Written informed consent was obtained from the patient involved in this study.

### P17 Identification of potent allergens in 104 skin biopsies from allergic tattoo reactions

#### Ines Schreiver^1^, Jorgen Serup^2^, Mitra Sepehri^2^, Nadine Dreiack^1^, Nils Dommershausen^1^, Lisa-Marie Eschner^3^, Peter Laux^1^, Andreas Luch^1^

##### ^1^German Federal Institute for Risk Assessment, Berlin, Germany; ^2^Bispebjerg University Hospital, Copenhagen, Denmark; ^3^Freie Universität Berlin, Berlin, Germany

###### **Correspondence:** Ines Schreiver - Ines.Schreiver@bfr.bund.de

*Clinical and Translational Allergy* 2017, **7(Supple 4):**P17

**Background:** Acutely occurring allergies are amongst the most severe side effects related to the ingredients of tattoo inks. Since tattoo associated allergens cannot be removed from dermal layers of the skin without invasive surgical methods, they may cause a severe threat to the respective patients. In the literature, allergic reactions to tattoos are by far most common with red color shades. However, the chemical origin of these colors is usually not reported and analytical evidence of the pigments in the skin or inks provided by the tattoo is not been given either. In former years, mostly inorganic pigments like iron oxides were used for red and yellow shades, thus bearing the risk of intradermal exposure to sensitizing elements such as Ni, Cd, Mn and Co. In the last decades, the use of highly light-fast and color brilliant organic pigments started to dominate the market.

Since the pigments themselves are insoluble in water, they are generally considered biologically inert. The allergen is suggested to be a hapten formed in the skin over time, possibly associated with pigment metabolites or other breakdown products. It is known that allergies might be induced by sunlight exposure or laser irradiation suggesting that a hapten might also derive from chemical decomposition of the pigment. Yet, no specific haptens have been identified so far. As a prerequisite for future regulation of tattoo pigments, harmful substances that bear the risk of allergy formation or other adverse reactions have to be identified.

**Methods:** Here, we screened 104 skin biopsies of patients who have developed an allergy against their red to violet tattoo. Specimens were analyzed for potential sensitizing elements using inductive-coupled plasma mass spectrometry (ICP-MS). Organic pigments were identified by matrix-assisted laser desorption/ionization time-of flight (MALDI-ToF)-MS/MS.

**Results:** About half of the samples contained Cr, Ni or both elements. Organic pigments in the samples belonged to the azo and quinacridone family and can be traced back to five predominantly occurring pigments. The pigments themselves, their known decomposition products and extracts from sunlight simulation will be tested by the direct-peptide reactivity assay (DPRA) to identify the potentially sensitizing compounds.

**Conclusions:** As a long term goal, the reactive compounds should be patch-tested in patients to achieve an ultimate proof of the true kind of sensitizer(s).

### P18 Airborne contact dermatitis due to diphencyprone

#### Esozia Arroabarren, Catalina Vela, Marta Anda, Oscar Antonio Terry, Antonio Juan Rodriguez

##### Complejo Hospitalario de Navarra, Pamplona, Spain

###### **Correspondence:** Esozia Arroabarren - esoziaa@yahoo.es

*Clinical and Translational Allergy* 2017, **7(Supple 4):**P18

**Background:** Diphencyprone is a known contactant used for its sensitizing properties, for the treatment of *alopecia areata*. There have been a few reports of occupational allergy. We report 2 occupational cases of airborne dermatitis due to diphencyprone.

**Methods:** A 55-year and 40-year old women presented with a 1-year history of recurrent erythematous, itchy lesions in neck and ventral area of both arms. Both denied desquamation or residual lesions. Patient 1 was asymptomatic during the holidays. Both patients related the symptoms with the exposure to dyphencyprone (both in case of direct contact or if other people manipulated it). Both worked in the magistral formulation area of the same pharmacy. Patient 1 was a technical laboratory assistant and patient 2 was a pharmacist. Protective measures implemented before symptom on-set included: white labcoats, FFP2 masks, nitrile gloves and paper hats). Besides, dyphencyprone, initially handled outside the laminar flow chamber, was subsequently handled in the flow chamber. Allergy work-up consisted of skin prick test with a battery of inhalant and food allergens, an alkali resistance test and patch tests. Patch tests were performed with standard sensitizers (ICDRG) and a battery containing the drugs manufactured in the pharmacy (levothyroxine, hydrocortisone, triamcinolone acetonide, dyphencyprone, gentamicin, urea, salicylic acid, gomenol, metoxalene, cobletasol propionate, retinoic acid, cyclophosphamide, latanoprost, simple syrup [sacarose and water], Propylene glycol, 2 syrup bases, anhydrous *lanolin*, inert excipient for capsules, triethanolamine, lather).

**Results:** Both patients tested positive for dyphencyprone 0.1% *pet* (blisters), 0.01% *pet* (++) and 0.001% *pet* (+), and for nickel sulphate. Patient 1 also tested positive for primine (+). Patient 2 tested positive for peanut and *D. pteronyssinus*. Patients’ symptom improved but did not disappear with the mentioned measures. Therefore, single-use, disposable protective avoidance measures were advised to improve its efficacy. Both have remained asymptomatic since then.

**Conclusions:** We report 2 cases of occupational allergy to dyphencyprone who needed to extreme avoidance measures for symptom resolution. Extreme precaution should be encouraged to handlers and users during manipulation to avoid further damage. Protective measures against airborne exposure should include protective work clothes, facial masks and dyphencyprone manipulation in a flow hood.

**Consent to publish** Written informed consent was obtained from the patients involved in this study.

### P19 Propolis and thiomersal—important baseline patch test series allergens in Slovenia

#### Mojca Bizjak, Nissera Bajrovic, Mihaela Zidarn, Renato Eržen, Peter Kopac, Nika Lalek, Mariana Paula Rezelj, Mitja Košnik

##### University Clinic of Respiratory and Allergic Diseases Golnik, Golnik, Slovenia

###### **Correspondence:** Mojca Bizjak - mojca.bizjak@klinika-golnik.si

*Clinical and Translational Allergy* 2017, **7(Supple 4):**P19

**Background:** The current European baseline patch test series recommended by the European Environmental and Contact Dermatitis Research Group does not include propolis and thiomersal. According to the European Society of Contact Dermatitis guideline for diagnostic patch testing an allergen is suggested for inclusion in the baseline series when routine patch testing of patients with suspected contact dermatitis results in a proportion of contact allergy to the substance exceeding 0.5–1.0%, and when this allergen is ubiquitous and/or clinically highly relevant. Propolis is composed of beeswax, oils, pollen and other substances. Since propolis, colophonium, fragrance mix I (FMI), and *Myroxylon pereirae* have shared constituents cross-sensitization can occur. Our objective was to investigate whether propolis and thiomersal should remain in baseline patch test series in Slovenia.

**Methods:** Seven hundred and eighty-two patients with suspected contact dermatitis were routinely patch tested with our baseline patch test series allergens between January 2014 and December 2016. Propolis 10% in petrolatum and thiomersal 0.1% in petrolatum were included. Square plastic chambers on hypoallergenic tape were used. The patch tests were removed and read at D2 and again at D3 according to the International Contact Dermatitis Research Group recommendations. Positive patch test reactions fulfilled the criteria of at least a one plus (+) reaction at D3.

**Results:** A total of 782 patients were patch tested. Eleven patients (1.4%) had a positive patch test to propolis (8 females and 3 males). Out of these 11 patients, 5 (45.5%) were also sensitized to FMI, 8 (72.7%) to colophonium, and 5 (45.5%) to *Myroxylon pereirae*. None out of 11 propolis allergic patients reacted to propolis only. Positive patch test reactions to thiomersal were found in 26 out of 782 (3.3%) routinely patch-tested patients.

**Conclusions:** Propolis and thiomersal are important allergens and their inclusion in our baseline series is appropriate. Propolis is an ubiquitous allergen of local importance to Slovenia as a beekeeping nation. It has not yet been systematically determined whether an established contact allergy to propolis in our patients is attributable to cross-reactivity. A proportion of contact allergy to thiomersal exceeds 1.0% and it should therefore also be included in our baseline series.

## Poster discussion session. Topic 3: Contact dermatitis - Group B

### P20 Allergic contact dermatitis to aluminium salts in routine childhood vaccinations

#### Cathal Padraig O’Connor, Rosemarie Watson

##### Our Lady’s Children’s Hospital Crumlin, Dublin, Ireland

###### **Correspondence:** Cathal Padraig O’Connor - cathaloconnor@umail.ucc.ie

*Clinical and Translational Allergy* 2017, **7(Supple 4):**P20

**Background:** Childhood immunisations are an essential component of an effective public health strategy. Local side effects such as redness and induration are common, due to local inflammation or haematoma formation. Aluminium phosphate or aluminium hydroxide are adjuvants frequently added to vaccines that potentiate the immune responses to an antigen and modulate it towards the desired immune responses. Aluminium sensitisation and contact allergy can occur after routine immunisation. The cases of four patients who developed varying reactions to the aluminium components of routine childhood vaccines are presented.

**Methods:** Patient 1 developed an erythematous nodule at the site of Bacille Calmette Guerin inoculation which resolved over months with some residual hyperpigmentation. Patient 2 developed pruritus in the days following vaccination with the Measles Mumps Rubella (MMR) and Haemophilus influenza type B (HiB) immunisations at 13 months of age. Patient 3 developed a sterile abscess with subsequent induration and hyperpigmentation following the 6 in 1 (DTaP, IPV, HiB, Hep B) and Pneumococcal conjugate vaccine (PCV) at four months of age. Patient 4 developed a nodule on her thing subsequent to her MMR and PCV vaccinations, with delayed itch and hyperpigmentation several months after the vaccine.

**Results:** Patients were referred to various medical specialties including general practice, immunology, infectious diseases, dermatology, and general surgery. All patient received differing treatment regimens, including topical steroids. These patients are being followed up in light of the risk of antiperspirant allergy.

**Conclusions:** Allergy contact dermatitis to aluminium is uncommon as a complication of routine immunisation but must be considered in the differential diagnosis of post vaccination local reactions.

### P21 Unusual case of allergic contact dermatitis to textile dyes

#### Kotryna Linauskiene, Laura Malinauskiene

##### Vilnius University Hospital Santariškiu Klinikos, Center of Pulmonology and Allergology, Vilnius, Lithuania

###### **Correspondence:** Kotryna Linauskiene - kotryna.lin@gmail.com

*Clinical and Translational Allergy* 2017, **7(Supple 4):**P21

**Background:** Typical allergic contact dermatitis from textile dyes presents as acute or subacute dermatitis at sites of friction and in major skin folds. However, patients may present a varied clinical picture, including urticaria and diffuse pruritus. Here we present a case of an atypical contact dermatitis from azo disperse dyes.

**Case report:** A 40-year-old female with no history of atopic dermatitis developed a pruritic eruption all over her body. During two years he was consulted by several dermatologists and their diagnoses were prurigo and dermatographic urticaria. Initial examination revealed infiltrated erythematous papules with pronounced scratching marks on both arms, legs and abdomen. Despite topical corticosteroid, the lesions worsened and spread. Skin biopsy showed normal epidermis with lymphocytic infiltration of the dermis consistent with urticarial reaction. When asked, the patient indicated that she had a contact dermatitis form hair dyes 7 years ago but still dyes her hair despite experiencing signs of scalp dermatitis. She is always dressed in black synthetic clothes. Patch testing was performed with the European baseline series and was positive to p-phenylenediamine, textile dye mix 6.6%, thiuram mix, cobalt chloride, nickel chloride (all 3 +) and neomycin sulphate (2 +). Of these reactions present clinical relevance was established only to p-phenylenediamine and textile dye mix. Avoidance of dark clothes made from synthetic fibers was recommended.

**Conclusions:** The diagnosis of textile dye contact dermatitis is often delayed because of unusual clinical presentation, and it is usually after patch testing that clothing is identified as causative.

**Consent to publish** Written informed consent was obtained from the patient involved in this study.

### P22 Allergic contact dermatitis (ACD) by clobetasol propionate and nicotinell patches

#### Simoneta Hernández Reyes^1^, Sara Burillo Martínez^2^, Marta Prieto Barrios^2^, Ana María Delgado Marquez^2^, Javier Ortíz De Frutos^2^

##### ^1^Allergy Department, Hospital Clínico San Carlos, Madrid, Spain; ^2^Dermatology Department, Hospital 12 de Octubre, Madrid, Spain

###### **Correspondence:** Simoneta Hernández Reyes - simonetahr@gmail.com

*Clinical and Translational Allergy* 2017, **7(Supple 4):**P22

Withdrawn

### P23 Allergic contact dermatitis from calcipotriol

#### Liesbeth Gilissen, Sara Huygens, An Goossens

##### University Hospitals KULeuven, Leuven, Belgium

###### **Correspondence:** Liesbeth Gilissen -liesbeth.gilissen@uzleuven.be

*Clinical and Translational Allergy* 2017, **7(Supple 4):**P23

**Background:** Calcipotriol, a synthetic vitamin D analogue, is widely used for the topical treatment of psoriasis. It often causes irritation reactions, whereas allergic contact dermatitis is less common [1]. Six patients with allergic contact dermatitis from calcipotriol, of whom 2 men and 4 women, were seen in our tertiary patch test clinic between 1990 and 2016.

**Methods:** Patch tests were performed with the commercial preparation used by the patients (LEO Pharmaceutical Products, Ballerup, Denmark) and/or its ingredients, including calcipotriol (2 or 10 µg/ml in isopropyl alcohol). IQ Ultra® Chambers (Chemotechnique Diagnostics, Vellinge, Sweden) were used and readings were scored according to the ESCD patch-testing guideline [2].

**Results:** For all six patients, allergic contact dermatitis was confirmed by at least one positive patch-test reaction (Table 1). In all cases, the lesions improved following substitution of the therapy by topical corticosteroids and/or oral medication.

**Table 1:** Results

Patient	Lesion location	Patch test with calcipotriol 2 µg/ml	Patch test with calcipotriol 10 µg/ml	Patch test with the commercial preparation
F 10y	Hands	D2 −, D4 +	NT	NT
F 49y	Feet	D2 +, D4 ++	D2 +, D4 ++	NT
F 59y	Scalp	D2 ?, D4 +	NT	D2 +, D4 +
F 50y	Feet	D2 +, D4 ++	NT	NT
M 26y	Hands and feet	D2 −, D3 +	NT	D2 NR, D4 ++
M 32y	Hands and feet	D2 +, D3 +	NT	D2 +, D3 +

**Conclusions:** When topical treatment with calcipotriol fails to improve or even worsens the existing lesions, calcipotriol contact allergy should be ruled out. In order to avoid irritant patch test reactions, a concentration of 2 µg/ml in isopropyl alcohol is the most suitable, and in unclear cases, a repeated open application test (ROAT) should be performed [3]. According to the literature, patients sensitized to calcipotriol may tolerate topical therapy with other vitamin D analogues, in particular tacalcitol [4].

**References**

1. Fowler J. Allergic and irritant contact dermatitis to calcipotriol. Am J Contact Dermat. 1999;10:78–80.
2. Johansen JD, Aalto-Korte K, Agner T, et al. ESCD guideline for diagnostic patch testing—recommendations on best practice. Contact Dermat. 2015;73:195–221.
3. Frosch P, Rustemeyer T. Contact allergy to calcipotriol does exist. Contact Dermat. 1999;40:66–71.
4. Zollner T, Ochsendorf F, Hensel O, et al. Delayed-type reactivity to calcipotriol without cross-sensitization to tacalcitol. Contact Dermat. 1997;37:251–2.

### P24 “No angry back” revisited

#### Marléne Anne Inger Isaksson, Maria Nilsson

##### Department of Occupational and Environmental Dermatology, Skane University Hospital, Malmö, Sweden

###### **Correspondence:** Marléne Anne Inger Isaksson - marlene.isaksson@med.lu.se

*Clinical and Translational Allergy* 2017, **7(Supple 4):**P24

**Background:** The term ‘angry back syndrome’ (ABS) was coined by Mitchell in 1975. It was stated that a strong positive patch test reaction could create an ‘angry back’ which becomes hyper-reactive to other patch test challenges. Usually marginal irritants may become “positive”. A 74-year-old atopic male had grommets inserted bilaterally 15 years prior to presentation and due to secretion through the tubes a chronic otitis externa developed with intermittent severe itching. His hearing aids made the problem even worse. He was referred to us because of suspicion of contact allergy to the hearing aid.

**Methods:** Patch testing was carried out with the Swedish baseline series (based on the European baseline series but supplemented with other sensitizers), an extended baseline series, a corticosteroid series, the grommets “as is”, and as ethanol and acetone extracts. Patch tests were removed after 48 hrs and readings were performed on day (D)4 and D7.

**Results:** Positive reactions were noted to 18 different test preparations on D4, 7 of which were +++, and 5 ++. On D7 14 positive reactions were noted, none of which were +++ and 8 of which were ++. Additional positive tests were noted to 7 preparations, 5 of which were to corticosteroids, and one each to methylisothiazolinone and colophony. Hydrocortisone-17-butyrate was one of the positive tests (+).

**Conclusions:** In the present case only the 18 separate tests were positive with completely normal-looking skin between, speaking against ABS. Former clinical relevance was noted to *Myroxylon pereirae*, tixocortol pivalate, hydrocortisone, Amerchol L 101, and disperse dye mix. Colophony, aluminum, and hydrocortisone-17-butyrate were considered to have present relevance. The patient used a solution for his ears intermittently containing hydrocortisone-17-butyrate, which was considered the only culprit allergen regarding the chronic otitis externa. No relevance was found for gold, nickel, para-phenylenediamine, formaldehyde, cain mix II, methyldibromo glutaronitrile, diphenylguanidine, propylene glycol, carba mix, methylchloroisothiazolinone/methylisothiazolinone, methylchloroisothiazolinone, methylisothiazolinone, linalool oxidized, limonene oxidized, alclometasone dipropionate, diflorasone diacetate, flumetasone, fluticasone propionate, and methylprednisolone aceponate.

**Consent to publish** Written informed consent was obtained from the patient involved in this study.

### P25 Contact dermatitis to cosmetics—relevance of a cosmetic series

#### Bárbara Kong Cardoso, Cíntia Rito Cruz, Eulália Matos, Elza Tomaz, Filipe Inácio

##### Centro Hospitalar de Setúbal - Hospital de São Bernardo, Setúbal, Portugal

###### **Correspondence:** Bárbara Kong Cardoso - barbarakc@gmail.com

*Clinical and Translational Allergy* 2017, **7(Supple 4):**P25

**Background:** Cosmetics are a frequent cause of allergic contact dermatitis (ACD). Epicutaneous tests (ET) with the Standard European Series (SES) and non-standardized cosmetic series (CS) are used to identify the culprit cosmetic component. The prevalence rate of ACD to cosmetics varies with time and geographic location, most influenced by the allergenicity of cosmetic ingredients, a population’s increased use of cosmetics over time and accessibility of allergens to be used in patch testing. The aim of this study was to evaluate the frequency of sensitization to cosmetics’ components in patients with suspected ACD, as well as to determine the utility of the test series used in the diagnosis.

**Methods:** Retrospective study including patients with suspected and/or diagnosed contact allergy to cosmetics over a 3-year period. Data regarding the location of eczema lesions, positive tests and clinical relevance as well as probable cause and final diagnosis were collected.

**Results:** A total of 138 patients (83% female) with a mean age of 47 years were enrolled, 11 of them under 18. From those, face and hands/fingers were the most commonly affected areas. The symptoms were widespread in 24% of the patients. All the patients underwent ET with the SES, and 66 were also submitted to ET with a CS. Seventy two percent had at least one positive test to a cosmetic component, and 16% had 2 or more sensitizations. Fifty eight percent of the patients that underwent ET with the SES plus a CS did not have any positivity. Only 5 patients had a positive result to components of the CS. The most frequent sensitizations were: lyral (22%), fragrance mix (14%), p-phenylenediamine (11%) and methylisothiazolinone (9%). Eighty-eight percent of the sensitizations were found to have a current relevance, being of occupational cause in 8 patients.

**Conclusions:** ACD was more frequently associated with lyral, fragrance mix, p-phenylenediamine and methylisothiazolinone, and the relation with occupational contact dermatitis was discreet. The majority of the patients that underwent ET with the SES plus a CS did not have any positivity, and only 3.6% of the patients benefitted from ET with the CS. Thus, we may assume that there is little advantage in performing patch testing with SES and a CS in one session. We suggest performing first ET with the SES and eventually proceed to ET with a CS in case of a negative result despite of a high clinical suspicion.

## Poster discussion session. Topic 4: Drug allergy

### P26 Etoricoxib induced fixed drug eruption confirmed by patch testing

#### Inese Hauksson

##### Department of Occupational and Environmental Dermatology, Skåne University Hospital, Malmö, Malmö, Sweden

###### **Correspondence:** Inese Hauksson - Inese.Hauksson@skane.se

*Clinical and Translational Allergy* 2017, **7(Supple 4):**P26

Withdrawn

### P27 Chlorhexidine allergy: Could it become a hidden healthcare epidemic?

#### Nadine Marrouche^1^, Clive Grattan^2^

##### ^1^Norfolk and Norwich University Hospital, Norwich, United Kingdom; ^2^Guy’s and St Thomas’ NHS Foundation Trust, London, United Kingdom

###### **Correspondence:** Nadine Marrouche - nadine.marrouche@gmail.com

*Clinical and Translational Allergy* 2017, **7(Supple 4):**P27

**Background:** We present a patient with a confirmed IgE-mediated chlorhexidine allergy and highlight some of the diagnostic pitfalls in the work-up of suspected allergy to this substance.

**Methods:** An 81-year-old female patient presented with an acute urticarial reaction in recovery following a colposuspension under general anaesthetic. She was covered in an intensely itchy rash concentrated on the lower abdomen and upper inner thighs. She was treated with IV hydrocortisone and chlorphenamine. The rash settled after 4 hours. Allergy to latex was initially suspected. The patient had contact with latex gloves and a latex catheter throughout the procedure. She had received bupivacaine as a local anaesthetic in addition to induction agents, a muscle relaxant, and antibiotics. The pelvic area had been prepared pre-operatively with a chlorhexidine containing skin wash. A chlorhexidine-based cream was inserted as a vaginal pack post-operatively.

**Results:** A RAST to latex was negative but chlorhexidine was positive (0.53 kAU/litre). Skin prick tests to latex (standardized extract) and bupivacaine (0.25%) were negative and aqueous chlorhexidine (0.5%) was inconclusive. Intradermal testing to chlorhexidine at 1:2500 dilution resulted in an itchy 10 mm weal response.

**Conclusions:** Chlorhexidine is an antiseptic that is commonly used to disinfect the skin ahead of invasive procedures. Allergy to chlorhexidine is rare. The spectrum of hypersensitivity reactions is wide and includes IgE-mediated anaphylaxis. Allergy to chlorhexidine is important to recognise as exposure is extensive and not only limited to the health care setting. Chlorhexidine is found in a number of personal care products including toothpaste, mouthwash, and even some cosmetics. Allergy testing to chlorhexidine can be a challenge. Skin testing remains an important diagnostic tool albeit when the appropriate concentrations are used for testing. In our case, intradermal testing was done using a dilution of chlorhexidine 0.5% which was initially difficult to source. Moreover, it appears that the IgE antibody response to chlorhexidine decreases with time and RAST becomes less sensitive leading to potential false negative results*. This report should remind all healthcare professionals of an important potential hazard of this widely used antiseptic.

**Consent to publish** Written informed consent was obtained from the patient involved in this study.

**Reference**

- Garvey LH, et al. IgE-mediated allergy to chlorhexidine. J Allergy Clin Immunol. 2007;120(2):409–15.

### P28 Widespread erythema following the use of a transdermal therapeutic system containing rivastigmine

#### Liesbeth Gilissen, Sara Huygens, An Goossens

##### University Hospitals KULeuven, Leuven, Belgium

###### **Correspondence:** Liesbeth Gilissen - liesbeth.gilissen@uzleuven.b

*Clinical and Translational Allergy* 2017, **7(Supple 4):**P28

Withdrawn

### P29 Ciprofloxacine induced Steven-Johnson syndrome—a case report

#### Sonila Dauti^1^, Esmeralda Shehu^2^

##### ^1^Hospital of Kavaja, Kavaja, Albania; ^2^Regional Hospital of Durres, Durres, Albania

###### **Correspondence:** Sonila Dauti - sonila_dauti@yahoo.com

*Clinical and Translational Allergy* 2017, **7(Supple 4):**P29

**Background:** Steven-Johnson Syndrome (SJS) is a serious life-threatening adverse reaction most commonly to drugs, including antibiotics. Ciprofloxacine is an antibiotic generally used for urinary tract infections.

**Methods:** We report the case of a 61-year old male who was on treatment with Ciprofloxacine for a urinary tract infection. He developed a Steven-Johnson syndrome on the third day of treatment. He was hospitalized at the allergy department at the fifth day of the treatment with fever, diffused maculo-papular rash, target-like lesions and blisters and mucous membrane erosions of ocular, oral and genital mucosa along with photofobia and difficulty of swallowing.

**Results:** All laboratory findings resulted within normal ranges except for a slightly elevated leukocytosis. The patient resulted negative to HIV, HBV, HCV, Chlamydia and Mycoplasma infections. He was treated mainly symptomatically with systemic corticosteroids, oral antihistamines, antiseptic compresses, mouthwashes and topical anesthetics for oral lesions and topical treatment with steroid and artificial teers for the ocular surface. The patient recovered completely on day 12, after discontinuing of ciprofloxacine and symptomatic treatment. Six weeks after dismission from the hospital we performed patch test with ciprofloxacine and the result was negative. We didn’t perform a challenge test with ciprofloxacine because of the severe adverse reaction he experienced before.

**Conclusions:** Drug regimen with ciprofloxacine and other fluorquinolones should be monitored carefully to prevent severe adverse cutaneous drug reactions that involve the skin and mucous membranes and interrupt immediately the therapy if Steven-Johnson Syndrome is suspected.

**Consent to publish** Written informed consent was obtained from the patient involved in this study.

### P30 An uncommon cause of maculopapular rash

#### Katharina Moritz, Tamar Kinaciyan

##### DIAID, Department of Dermatology Medical University of Vienna, Vienna, Austria

###### **Correspondence:** Tamar Kinaciyan - tamar.kinaciyan@meduniwien.ac.at

*Clinical and Translational Allergy* 2017, **7(Supple 4):**P30

**Background:** A young lady suffering from common cold treated herself with a combination preparation of 500 mg acetylsalicylic acid (ASA) and 30 mg pseudoephedrine hydrochloride dissolved in a glass of water which leads to a quick amelioration of nasal congestion and cough. The following day, she developed a generalized itchy maculopapular rash. After discontinuation of the medication and application of topical steroids twice daily, the rush disappeared within 5 days. She was presented at our outpatient clinic for allergologic work-up.

**Methods:** Skin Testing (prick-, intradermal- and patch-test) was performed with an ASA mono-preparation for i.v. use, the ASA - pseudoephedrine combination preparation and etilefrine mono-preparation for i.v. use.

**Results:** Prick- and intradermal tests resulted negative for all tested medications. Patch testing revealed a similar strongly positive reaction to the combination preparation and etilefrine after 24 h whereas ASA mono-preparation test remained negative.

**Conclusions:** Pseudoephedrine is a widely used vasoconstrictive sympathomimetic agent. It is used topically as a mydriatic drug in ophthalmology or as decongestant nose drops. As systemic medication t is used for the treatment of low blood pressure or paroxysmal atrial tachycardia and in combination with non-steroidal anti-inflammatory drugs for the treatment of common cold. Allergic reactions to pseudoephedrine are very rare except for allergic contact dermatitis upon external use in ophthalmology. There are few cases of a rash with joint swelling, a toxic shock syndrome and fixed drug eruptions due to pseudoephedrine described. Little is known about cross-reactivity between different sympathomimetics other than pseudoephedrine, and ephedrine which are structurally very similar. We could hereby demonstrate that there is a cross-sensitivity between pseudoephedrine and etilefrine as well.

## Poster discussion session. Topic 5: Experimental

### P31 The skin reservoir model: a novel approach to estimate recovery of large biomarkers from human skin using microdialysis

#### Katrine Yderstræde Baumann^1^, Per Stahl Skov^2^, Anders Woetmann^3^, Sally Dabelsteen^3^, Sidsel Falkencrone^2^

##### ^1^RefLab ApS and University of Copenhagen, Copenhagen, Denmark; ^2^RefLab ApS, Copenhagen, Denmark; ^3^University of Copenhagen, Copenhagen, Denmark

###### **Correspondence:** Katrine Yderstræde Baumann - kybaumann@gmail.com

*Clinical and Translational Allergy* 2017, **7(Supple 4):**P31

**Background:** Microdialysis has been extensively used to sample small unbound molecules from various tissues including the skin. However, sampling of larger molecules such as cytokines is more complicated, why in vitro assessment of sampling feasibility is crucial for every molecule of interest. Relative recoveries, defined as the fraction of the periprobe concentration collected in the dialysate, have traditionally been estimated in vitro by sampling from a reference solution, yet this approach pose a problem for probes with high molecular weight cut-offs (MWCO), as the larger pores cause leakage into the surrounding fluid, which often lead to no fluid recovery at all. Furthermore, several molecules are known to bind extracellular matrix components leading to a decreased recovery, which is not detected in in vitro reference solution setups. To overcome this, we suggest a novel approach using inert intact human ex vivo skin to estimate relative recoveries with cutaneous microdialysis.

**Methods:** Skin from three donors was obtained and frozen at − 20 °C prior to use. Microdialysis probes (3000 kDa MWCO) were inserted in thawed skin specimens (n = 3). IL-1α, IL-6, IL-17, and TNF-α in three concentrations, or a vehicle control (perfusate), were injected around three replicate probes in each donor (n = 12 probes per donor) mimicking a reservoir around the probes. Probes were perfused with a solution of 1% human albumin in Ringer-lactate at 0.8 µl/min and dialysates were collected for 2 hours. Background levels in ex vivo skin and relative recoveries were determined by quantifying dialysate concentrations using ELISA.

**Results:** The ultrafiltration observed in previous in vitro setups was markedly reduced in the skin reservoir model, as inserted probes yielded 80–110% of the expected volume output. Average relative recoveries of cytokines were independent of periprobe concentrations and quantified as 15.2% (IL-1α), 9.3% (IL-6), 10.4% (IL-17), and 6.3% (TNF-α). The coefficient of variation was ~ 30% and independent of skin donor. IL-1α was the only cytokine with background levels above detection limits.

**Conclusions:** The skin reservoir model is a novel approach to estimate relative recoveries of large unbound molecules by cutaneous microdialysis. This setup diminished problems with probe leakage seen in previous in vitro approaches. Furthermore, this model closer resembles an in vivo cutaneous environment, why we believe the relative recoveries obtained more accurately represents potential in vivo recoveries.

### P32 TRPV channels and post-burn pruritus

#### Hye One Kim, Yong Won Choi, Jee Hee Son, Yong Se Cho, Bo Young Chung, Chun Wook Park

##### Department of Dermatology, Kangnam Sacred Heart Hospital, Seoul, South Korea

###### **Correspondence:** Hye One Kim - hyeonekim@gmail.com

*Clinical and Translational Allergy* 2017, **7(Supple 4):**P32

**Background:** Post-burn pruritus is a common distressing sequela of burn wounds. Empirical antipruritic treatment often fails to have a satisfactory outcome because the mechanism has not been fully elucidated. Transient receptor potential (TRP) channels are considered to be related to pathway of pruritus.

**Methods:** Sixty-five burn patients with (n = 40) or without (n = 25) pruritus were investigated, including skin biopsies. Keratinocytes and fibroblasts from those samples were separated. Immunohistochemical staining for TRPV3 and TRPA1; and immunofluorescence staining for TSLP, TSLPR, loricrin, involucrin, -SMA, and TGF-, were performed on samples of burn scars and normal skin. Real-time PCR and western blotting of TRPV3, TRPA1, PAR2 NK1R, TSLP, and TSLPR were done. We also measured intracellular Ca^2+^ levels in keratinocytes from scars with or without pruritus, following TRPV3 activation and blocking, and measured the effects of PAR2 agonist on TRPV3 function. Expressions of TSLP after TRPV3 activation in keratinocytes were measured by western blotting and real-time PCR.

**Results:** In immunohistochemical and immunofluorescence staining, TRPV3, TSLP, and TSLPR stained more intensely the epidermis of the burn scars of post-burn-pruritus patients, than that of non-pruritic-burn patients. Real time-PCR showed that mRNA of TRPV3 and TSLP were significantly more abundant in keratinocytes from pruritic burn scars than in keratinocytes from non-pruritic burn scars. In addition, mRNA and protein levels of PAR2, NK1R, TSLP, and TSLPR were also significantly increased in pruritic burn scars. With TRPV 3 activation, intracellular Ca^2+^ concentrations were more significantly increased in keratinocytes from pruritic burn scars than in those from non-pruritic ones. In keratinocytes from pruritic burn scars, PAR2 activation markedly potentiated opening of TRPV3 channels. TRPV3 activation itself resulted in little increase of Ca^2+^ influx with PAR2 inhibition in keratinocytes. In keratinocytes from all samples, PLC-β, PKA, PKCs, and PKD inhibitor markedly reduced intracellular Ca^2+^ level by TRPV3 activation, as well as by PAR2 activation. TRPV3 activation also increased mRNA and protein expression of TSLP in keratinocytes.

**Conclusions:** We confirmed that TRPV3 of keratinocytes and PAR2, NK1R, TSLP, and TSLPR were highly expressed in pruritic burn scars. In addition, it seemed that PAR2 sensitized TRPV3 channels with PKA, PKC, PKD signaling pathways. It also seemed that TRPV3 activation induced TSLP expression.

### P33 Skin inflammation of K5-Ikzf1-EGFP transgenic mice

#### Mayumi Ueta, Junji Hamuro, Shigeru Kinoshita

##### Kyoto Prefectural University of Medicine, Kyoto, Japan

###### **Correspondence:** Mayumi Ueta - mueta@koto.kpu-m.ac.jp

*Clinical and Translational Allergy* 2017, **7(Supple 4):**P33

**Background:** We previously reported that Cold medicine related Stevens-Johnson syndrome/toxic epidermal necrolysis was significantly associated with Ikaros Family Zinc Finger 1 (*IKZF1*) polymorphisms. We investigated the function of *IKZF1 gene* in epidermis.

**Methods:** We generated K5-Ikzf1-EGFP transgenic mice (Ikzf1 Tg) by introducing the Ik1 isoform into cells expressing keratin 5, which is expressed in epithelial tissues such as the epidermis and then examined them histologically. Moreover, Ikzf1 Tg were induced allergic contact dermatitis.

**Results:** Ikzf1 Tg developed dermatitis with hair loss. Histological analysis also showed dermatitis in the transgenic mice; we observed mild thickening of the epidermis and moderate infiltration of the dermis by inflammatory cells. In contact dermatitis model, inflammatory infiltrates in the skin of Ikzf1 Tg were significantly increased compared with wild type.

**Conclusions:** Ikaros might participate in skin inflammation.

### P34 Topical cidofovir for recalcitrant viral skin lesions in Jacobsen syndrome

#### Cathal Padraig O’Connor, Ronan Leahy, Alan Irvine

##### Our Lady’s Children’s Hospital Crumlin, Dublin, Ireland

###### **Correspondence:** Cathal Padraig O’Connor - cathaloconnor@umail.ucc.ie

*Clinical and Translational Allergy* 2017, **7(Supple 4):**P34

**Background:** Two cases of Jacobsen syndrome are presented to highlight the association with intractable cutaneous skin infections. Jacobsen syndrome is a rare congenital disorder caused by a deletion in 11q24.1. It is characterised by intellectual difficulties, a distinctive facies, and various medical problems including cardiac defects, bleeding diathesis (Paris Trousseau syndrome), and short stature. Combined immunodeficiency has recently been described in the syndrome. Diffuse cutaneous viral infections have not been widely reported.

**Methods:** Patient 1 was diagnosed with Jacobsen syndrome at the age of 4 years, with a history of ventricular septal defect, bilateral duplex kidneys, recurrent epistaxis, and developmental delay. She was referred to our immunology colleagues with a history of recurrent ear, chest, and skin infections, including a breast abscess. Extensive molluscum contagiosum was noted on initial assessment. She had a history of recurrent herpes labialis and viral warts. Patient 2 was diagnosed with Jacobsen syndrome at the age of 18 months with a history of ventricular septal defect, thrombocytopaenia, and developmental delay. He was referred to our immunology colleagues in light of potential immunodeficiency. He had a history of recurrent viral warts on his hands but no other recurrent infections.

**Results:** Patient 1 was prescribed valaciclovir and co-trimoxazole prophylaxis. Topical cantharone was applied to the molluscoid lesions, with a very slow response. Treatment with topical cidofovir was initiated with excellent effect. Patient 2 was prescribed topical imiquimod 5%, with very poor response. Treatment with topical cidofovir has been initiated, with response awaited.

**Conclusions:** Jacobsen syndrome is a rare chromosomal abnormality associated with dysmorphic features and variable degrees of immunodeficiency. Topical cantharone and imiquimod may not be useful in treating viral cutaneous infections in these patients. Cidofovir inhibits viral replication by selectively inhibiting viral DNA polymerases. Topical cidofovir, although extremely expensive, may be required for recalcitrant lesions.

**Consent to publish** Written informed consent was obtained from the patients involved in this study.

## Poster discussion session. Topic 6: Miscellanous cutanoeus and allergic disorders

### P35 Autoinflammatory syndrome presenting as diffuse papular rash in infancy

#### Cathal Padraig O’Connor, Alan Irvine, Grainne O’Regan

##### Our Lady’s Children’s Hospital Crumlin, Dublin, Ireland

###### **Correspondence:** Cathal Padraig O’Connor - cathaloconnor@umail.ucc.ie

*Clinical and Translational Allergy* 2017, **7(Supple 4):**P35

**Background:** A six month old girl was referred to the dermatology outpatient department with a brown papular perifollicular rash on her trunk, face, and all four limbs, which began at three months of age. It was noted to spread from the site of her BCG vaccination, administered in the first week of life. She had no history of recurrent fever, uveitis, arthritis, or cranial nerve palsies. On examination, there were no mouth ulcers, no lymphadenopathy, no organomegaly, and no camptodactyly. Ophthalmology review found no evidence of intraocular inflammation. A skin biopsy demonstrated a granulomatous folliculitis, raising the possibility of a sarcoid-related condition. Her chest x ray was normal. Genetic investigations revealed a heterozygous mutation in the CARD15/NOD2 gene, in keeping with Blau syndrome. Her other investigations are outlined below. On further review at 12 months of age, her rash was still present but had faded in colour and become macular. She had developed recurrent fever and her hands were noted to have become oedematous, but not tender on palpation. There had been three episodes of eye redness.

**Methods:** On subsequent reviews, she had developed camptodactyly and arthritis. Immunomodulatory therapy was initiated with adalimumab, with an initial good response. Due to a flare of arthritis, methotrexate was added to optimise care.

**Results:** Bloods - FBC: Platelets 560(150–400), Lymphocytes 16.8(3.0–13.5); ESR 12(1–9), CRP < 4 (< 4); Calcium normal; ACE 71(8–65); Renal function normal; Liver function normal, Chest X Ray – Normal, Skin Biopsy - Granulomatous Folliculitis, Genetics - Heterozygous c.1808A > Gp.(His603Arg) mutation in CARD15/NOD2 on chromosome 16q12. This variant has not reported before in the literature and has not been detected in control population databases. Parental genetic testing is awaited, although mutations in Blau syndrome are often de novo.

**Conclusions:** Blau syndrome is associated with autosomal dominant, gain of function, mutations in the CARD15/NOD2 gene. Mutations in CARD15 cause an activation of nuclear kappa factor beta, which in turn upregulates proinflammatory cytokine transcription. Blau syndrome is phenotypically characterised by the triad of granulomatous dermatitis, polyarthritis, and uveitis. Immunosuppressive regimens for Blau syndrome have included systemic corticosteroids, methotrexate, tumour necrosis factor inhibition, and interleukin-1 inhibition.

**Consent to publish** Written informed consent was obtained from the guardians of the patient involved in this study.

### P36 Orofacial and napkin dermatitis as manifestation of zinc deficiency in premature neonate

#### Cathal Padraig O’Connor, Rosemarie Watson, Fiona Browne

##### Our Lady’s Children’s Hospital Crumlin, Dublin, Ireland

###### **Correspondence:** Cathal Padraig O’Connor - cathaloconnor@umail.ucc.ie

*Clinical and Translational Allergy* 2017, **7(Supple 4):**P36

**Background:** A 19 week old infant (five weeks corrected) presented to the emergency department of our hospital with an orofacial and perineal rash of one week duration. He had a background of extreme prematurity, (25 weeks and 5 days gestation) and suspected necrotising enterocolitis. He had no history of parenteral nutrition, and no family history of zinc deficiency or cystic fibrosis. He was exclusively breastfed since birth. One week prior to presentation he developed flesh-coloured and inflamed papules on his chin associated with scale. They spread over days to involve his cheeks, peinasal area, and suprapinnar fissures. The lesions then became red and crusted over. Simultaneously, he had developed a symmetrical erosive dermatitis in his napkin area. There was associated ‘peeling paint’ desquamation and scrotal oedema. There was a sharp demarcation to the abnormal areas of the skin, with the neck, trunk, and limbs markedly spared. The nails were normal and there was no loss of hair. He had a history of loose, light green stools since meconium had been passed in early life. He was otherwise healthy, feeding well, and apyrexial.

**Methods:** Treatment was initiated by the general paediatric team with intravenous antibiotic and antiviral therapy, with no improvement. Of note, a topical barrier ointment containing zinc oxide was used on the napkin area but not the face, and was associated with a significant improvement in that area. Following consultation with the dermatology team, a zinc level and skin biopsy were performed. Zinc supplementation at 1 mg/kg twice daily was empirically initiated. A dramatic and rapid improvement was noted within 48 hours.

**Results:**

- Zinc Level: 0.8 (6.6–13.9)
- Alkaline Phosphatase 200 (60–580)
- Protein 47 (60–80)
- Albumin 35 (36–50)
- Skin biopsy – striking confluent parakeratosis, absent granular layer, mild epidermal hyperplasia, consistent with zinc deficiency
- Maternal serum and breastmilk zinc – awaited

**Conclusions:** Zinc is a cofactor for many enzymes and is transferred via the placenta during the third trimester of pregnancy. Risk factors for zinc deficiency include premature birth, male gender, vegan/vegetarian Mother, and low albumin. Sign of zinc deficiency in infancy include periorofacial and acral dermatitis, diarrhea, behavioural change, and neurological disturbance. Our patient was a premature, breastfed, male infant with diarrhoea and orofacial and napkin dermatitis who had a remarkable recovery post zinc supplementation.

**Consent to publish** Written informed consent was obtained from the guardians of the patient involved in this study.

## Saturday, 29 April 2017

**Selected oral abstract presentations**

### O13 Does activated mast cells contribute to the metabolism of subcutaneous administered drugs?

#### Sidsel Falkencrone^1^, Per Stahl Skov^1^, Torsten Michael Reinheimer^2^, Anders Sonesson^2^

##### ^1^RefLab ApS, København ø, Denmark; ^2^Ferring Pharmaceuticals A/S, København S, Denmark

###### **Correspondence:** Per Stahl Skov - pss@reflab.dk

*Clinical and Translational Allergy* 2017, **7(Supple 4):**O13

**Background:** Mast cells are known to contain large amounts of mediators and proteases that are released upon activation during infections or allergy. It is however not know to what degree these mediators affects the metabolic rate of drugs in the skin. In order to investigate if mediators from activated mast cells contribute to the metabolism of subcutaneous (sc) administered drugs, intact ex vivo human skin was activated with/without codeine, and metabolism of a peptide drug, known to undergo rapid degradation after sc administration in a previous phase I study, was investigated. Metabolism was estimated over 21 hours by cutaneous microdialysis, which can be applied to investigate immunological reactions in the skin by sampling low and high molecular weight biomarkers as well as pre-clinical drugs from the extracellular compartment.

**Methods:** Healthy abdominal ex vivo skin was obtained after cosmetic surgery with full ethical consent. Microdialysis probes with a molecular cut-off of 300 kDa were inserted 2 cm intradermally into the skin, which was injected with a mast cell degranulator (codeine), or a buffer control. The peptide drug was subsequent administrated sc, and dialysates were collected over a period of 21 hours and analyzed by liquid chromatography with tandem mass spectrometry detection after desalting of the samples. Skin was incubated on Transwell membranes with a skin optimized medium and kept at 37 °C in a cell incubator until point of sampling.

**Results:** The results demonstrate a rapid degradation of the peptide drug in the skin forming a known metabolite. Degradation of the metabolite was followed during 21 hours and found to be slower than that of the parent peptide drug. However, no difference between metabolism in skin sites injected with codeine and sites injected with the buffer control could be detected. The rate of metabolism in the ex vivo skin was on par with the previous clinical observations.

**Conclusions:** The experiments demonstrated that while ex vivo skin did metabolize the peptide drug candidate, activation of in situ mast cells with subsequent release of proteases did not appear to increase the metabolic rate of this particular drug. However, the ex vivo skin metabolism model has proven to be a very useful model for the study of drug metabolism and ought to be considered for investigation of metabolism of other drugs or even the fate of sc administered allergens for allergy testing.

### O14 Occupational allergy due to acrylates: a case series

#### Catalina Vela, Esozia Arroabarren, Oscar Antonio Terry, Marta Anda, Antonio Juan Rodriguez

##### Complejo Hospitalario de Navarra, Pamplona, Spain

###### **Correspondence:** Esozia Arroabarren - esoziaa@yahoo.es

*Clinical and Translational Allergy* 2017, **7(Supple 4):**O14

**Background:** Methacrylates’ role in occupational allergy (OA) is well known among dentists and beauticians. Data regarding other exposures is scarce.

**Methods:** We report 20 patients with contact dermatitis due to acrylates and their features.

**Results:** Median age: 38 years (24–59y). Sex: Male: 50%. OA was suspected in 18 (90%). Professions: Beauticians: 6 cases; car assemblers (CA): 4; wind turbine generator assemblers (WEGa): 4; one dentist; a solar panel handler; a silicon handler; a printer worker, a dentist’s patient and an artificial nail user. Fifteen (75%) reported hand eczema, 4: face lesions. The dentists’ patient referred burning mouth symptoms. Patch tests were performed with a commercially available methacrylate group (MG) (Bial Aristegui, containing 10 methacrylates) in 14 (70%). Patch tests were also performed with suspected elicitors according to individual exposure (100%). All the patients tested with MG had positive results. All the beauticians tested positive for ethylene glycol dimethacrylate, 5 (80%) also to hydroxymethyl acrylate, and 1(16.6%) to triethylene glycol dimetacrylate. Three CA tested positive for ethyleneglycol methacrylate and hydroxyethyl methacrylate (HEMA), 1 for triethylene glycol dymetacrylate and 1 only to hydroxypropyl methacrylate. The dentist tested positive for ethylene glycol dimethacrylate, and HEMA. Silicon handler tested positive for hydroxyl methyl methacrylate. One of the WEGa tested positive for hydroxyl methyl methacrylate. Patients not tested with MG: Three WEGa and solar panel assembler tested positive for industrial adhesives containing acrylates (source of exposition). Burning mouth syndrome patient tested positive ethylene glycol dimethacrylate. Nail user tested positive for cyane acrylate and the printer for acrylate containing inks and varnishes. In all the OA suspected cases, patch tests were positive with acrylate containing substances delivered by the patients.

**Conclusions:** Our results suggest acrylate allergy should be suspected in other occupations. Ethyleneglycol methacrylate and hydroxyethyl methacrylate were the most frequently detected among the MG, present in 93% of the tested patients.

### O15 Detection and quantification of hand eczema lesions on images by machine learning

#### Stephan Schnürle^1^, Thomas Koller^1^, Marc Pouly^1^, Tim Vor Der Brück^1^, Jörg Hofstetter^1^, Florian Anzengruber^2^, Vahid Djamei^3^, Alexander A. Navarini^2^

##### ^1^Hochschule Luzern, Luzern, Switzerland; ^2^University Hospital of Zurich, Department of Dermatology, Zürich, Switzerland; ^3^Swiss4ward GmbH, Zürich, Switzerland

###### **Correspondence:** Alexander A. Navarini - alexander.navarini@usz.ch

*Clinical and Translational Allergy* 2017, **7(Supple 4):**O15

**Background:** Hand eczema (HE) is the third most frequent work-related disease. It occurs in about 10% of the working population. Immense loss of productivity results due to sick leave, which on average has a duration of 12 weeks. HE produces well-defined visible clinical skin changes. Early detection of hand eczema by the presence of such lesions could allow treatment before the disease becomes clinically relevant. Here we have constructed a machine learning approach to achieve this goal.

**Methods:** 30 dermatologists were invited to each mark the lesions of 48 images of hand eczema of various intensities using a java-based web platform (‘SkinAppWeb’). The markings were then used to calculate averages of coverage, and heat-maps were constructed to indicate the confidence of clinically evident HE at each spot. A supervised machine learning algorithm was then trained with these images (‘SkinApp’) and run on a separate batch of images. Using the heat-maps, sensitivity, precision and specificity scores can be adapted depending on the clinical situation.

**Results:** SkinApp resulted in detection of HE with an accuracy of 89.3% for the front side of the hands and 88.7% for the back using a ninefold cross validation scheme. The F1 scores (harmonic mean between sensitivity and specificity) are 58.6 and 44.1% respectively on standardized clinical pictures where at least 50% of the experts have marked HE.

**Conclusions:** Machine learning algorithm was able to detect features of HE on simple JPG pictures. Paired with comparisons of HE severity over time, this approach could allow for automated follow-up of HE patients and, once clinically validated, replace manual HE grading scores such as the HECSI score, which takes 10 min to calculate. Some difficulties were encountered in photos with uncontrolled angles of shadows and variable intensities of ambient or direct light. Thus, a portable box housing lighting and photoequipment (‘photobox’) was created to allow for stable lighting conditions. This opens the way for further validation of these techniques with the ultimate goal of making a screening device available for companies with high rates of HE and resulting work loss.

### O16 Patch testing in the diagnosis of hand eczema

#### Cíntia Rito Cruz, Bárbara Kong Cardoso, Rute Pereia Dos Reis, Eulália Matos, Elza Tomaz, Filipe Inácio

##### Centro Hospitalar de Setúbal - Hospital de São Bernardo, Setúbal, Portugal

###### **Correspondence:** Cíntia Rito Cruz - cintiacruz87@gmail.com

*Clinical and Translational Allergy* 2017, **7(Supple 4):**O16

**Background:** Hand eczema is a common condition, being allergic contact dermatitis (CD) an important cause. However, several studies point out the irritant CD as the most prevalent form of hand eczema. Clinical presentation alone does not allow to discriminate between the two conditions. The aim of this study was to detect the prevalence of allergic CD in patients with hand eczema as well as the implicated allergens.

**Methods:** We included all the patients with hand eczema who underwent epicutaneous tests (ET) with the Standard European Series (SES) and other non-standardized series over a 3-year period. Data were then collected regarding professional occupation, location of the lesions, results of the ET, relevance of the sensitization found, its probable cause and the final diagnosis.

**Results:** A total of 80 patients (71% female) with a mean age of 45 years were enrolled, four of them under 18. Fifty nine percent of the patients had a positive result (67% in women vs. 39% in men). The mean number of positive results per person was 2.3. Forty three percent of the patients also had eczema in other locations, with a slightly higher prevalence of positive results (65%) comparing to the patients with only hand eczema (54%). The most frequent sensitizations were: cosmetics (37%), metals (30%) and rubber additives (11%). In 43% of the patients the allergen identified was relevant. Out of the patients with positive results, 23% had a work-related condition, being the most common: hairdresser/beautician, manual labour and food preparation. In the patients with negative ET, the final diagnosis was irritant CD (61%), atopic dermatitis (3%) and dyshidrotic eczema (9%). A final diagnosis was not possible in 24%.

**Conclusions:** Our study suggests the importance of ruling out allergic CD in hand eczemas, as we obtained positivities in more than half of the patients. Allergic CD was almost equally found in patients with eczema in multiple locations. The proportion of occupation-related eczema was significant, similar to other studies results that identify hand dermatitis as a frequent occupational disease. We emphasize the difficulty in classifying hand eczema as we had a high proportion of patients without a final diagnosis.

